# A programmable *Aeromonas* chassis, AMAX2, for advanced biomanufacturing

**DOI:** 10.1128/aem.00231-26

**Published:** 2026-05-18

**Authors:** Ming-Xuan Tang, Yu-Zhao Liu, Jia-Xin Liang, Ruo-Lin Huang, Xuepiao Pu, Chen-Chen Liang, Zi-Yu Tang, Tong-Tong Pei, Ya-Jie Zhao, Hao-Yu Zheng, Tingting Zhang, Zixian Wu, Ying An, Xiaoye Liang, Xue Liu, Tao Dong

**Affiliations:** 1 School of Life Sciences, Guangming Advanced Research Institute, Southern University of Science and Technology 656360, Shenzhen, Guangdong, China; 2 State Key Laboratory of Microbial Metabolism, Joint International Research Laboratory of Metabolic and Developmental Sciences, School of Life Sciences and Biotechnology, Shanghai Jiao Tong University 553742 , Shanghai, China; 3 Department of Pathogen Biology, Base for International Science and Technology Cooperation: Carson Cancer Stem Cell Vaccines R&D Center, International Cancer Center, Shenzhen University Medical School 617011, Shenzhen, Guangdong, China; 4 Guangdong Key Laboratory for Biomedical Measurements and Ultrasound Imaging, National-Regional Key Technology Engineering Laboratory for Medical Ultrasound, School of Biomedical Engineering, Shenzhen University Medical School 620599 , Shenzhen, Guangdong, China; Chalmers tekniska hogskola AB, Gothenburg, Sweden

**Keywords:** protein expression and delivery, improved biosafety, minicell, surface display, T6SS

## Abstract

Synthetic biology increasingly demands engineered bacterial chassis that combine robust biosynthesis with tailored functionalities. We previously developed AMAX, an *Aeromonas*-derived chassis with fast growth, high GC content, and exceptional recombinant protein yield. Here, we report AMAX2, a next-generation chassis generated by deleting additional virulence-associated and nonessential genes in AMAX to enhance biosafety while preserving robust growth and protein expression. AMAX2 supports diverse inducible systems, multiple plasmid types, and tRNA supplementation to enable rare codon-enriched gene expression. Genome-wide CRISPRi-seq identified 153 core and 289 conditionally essential genes. Beyond expression, AMAX2 can be engineered for the production of DNA-free minicells, surface display for targeted protein decoration via SpyCatcher-SpyTag, and protein translocation into specific target cells via nanobody display and a plasmid-borne type VI secretion system. These features establish AMAX2 as a programmable chassis for advanced biomanufacturing. Comprehensive *in vitro* and *in vivo* assays showed that AMAX2 exhibits superior biosafety, with no detectable hemolysis, cytotoxicity, or pathogenicity in cellular and animal models. These multifunctional capabilities position AMAX2 as a versatile platform for industrial biomanufacturing and biotherapeutic development.

**IMPORTANCE:**

AMAX2 is a genetically programmable chassis that bridges industrial protein production and advanced functional applications, including targeted secretion, surface display, and minicellgeneration. Its unique combination of high performance, broad genetic compatibility, and multifunctionality provides a competitive alternative to existing platforms. By integrating genome-wide essential gene mapping and customizable delivery pathways, AMAX2 holds the potential for serving as a next-generation microbial platform in synthetic biology.

## INTRODUCTION

Bacterial chassis are central to synthetic biology, serving as the biosynthetic engines for producing recombinant proteins, metabolites, and other value-added products (1–3). An optimal chassis must integrate high productivity, robust stress tolerance, genetic tractability, and stringent biosafety with the flexibility to accommodate specialized functionalities. Increasingly, the role of a chassis extends beyond bulk protein expression to encompass modular systems for targeted recognition, metabolic engineering, and therapeutic applications (4–6).

We previously developed AMAX, an *Aeromonas*-derived high-GC (61.5%) chassis with several intrinsic advantages: rapid growth, high recombinant protein yield (target proteins reaching 60%–70% of total protein), broad compatibility with *Escherichia coli* vectors, and robust salt tolerance (7). While AMAX already serves as a high-performance expression host, next-generation chassis development requires further development with enhanced biosafety, expanded versatility, and precise genomic modulation to meet industrial, biomedical, and environmental standards.

Key engineering strategies for advanced chassis include genome-wide essential gene identification to guide precise genome reduction and modulation, and transplanting surface recognition and protein delivery modules to confer additional capabilities. CRISPR interference (CRISPRi) enables high-throughput functional screening to reveal genes critical for growth and biosynthesis (8–10). Surface display systems, including SpyCatcher-SpyTag and nanobody-antigen pairing, allow the presentation or recognition of antigens, ligands, or binding domains for targeted interactions (11, 12), while minicell production generates DNA-free, metabolically active vesicles ideal for safe delivery of therapeutic cargos (13, 14). Protein cargos can be delivered via dedicated secretion systems to further expand chassis functionality, enabling direct protein translocation into diverse cell types for antimicrobial, therapeutic, or signaling purposes (15, 16).

In this study, we report AMAX2, a derivative of AMAX generated by deleting additional virulence-associated and nonessential genes, resulting in enhanced biosafety without compromising protein production capacity. Genome-wide essential gene mapping via CRISPRi-seq revealed core gene functions. We demonstrate that AMAX2 can be functionally equipped with broad plasmid-borne induction systems, surface display modules, minicell generation, and engineered protein secretion. These multifunctional features position AMAX2 as a versatile, safe, and genetically tractable chassis for biotechnological applications.

## RESULTS

### AMAX2 genome features and protein production capabilities

**Fig 1:**
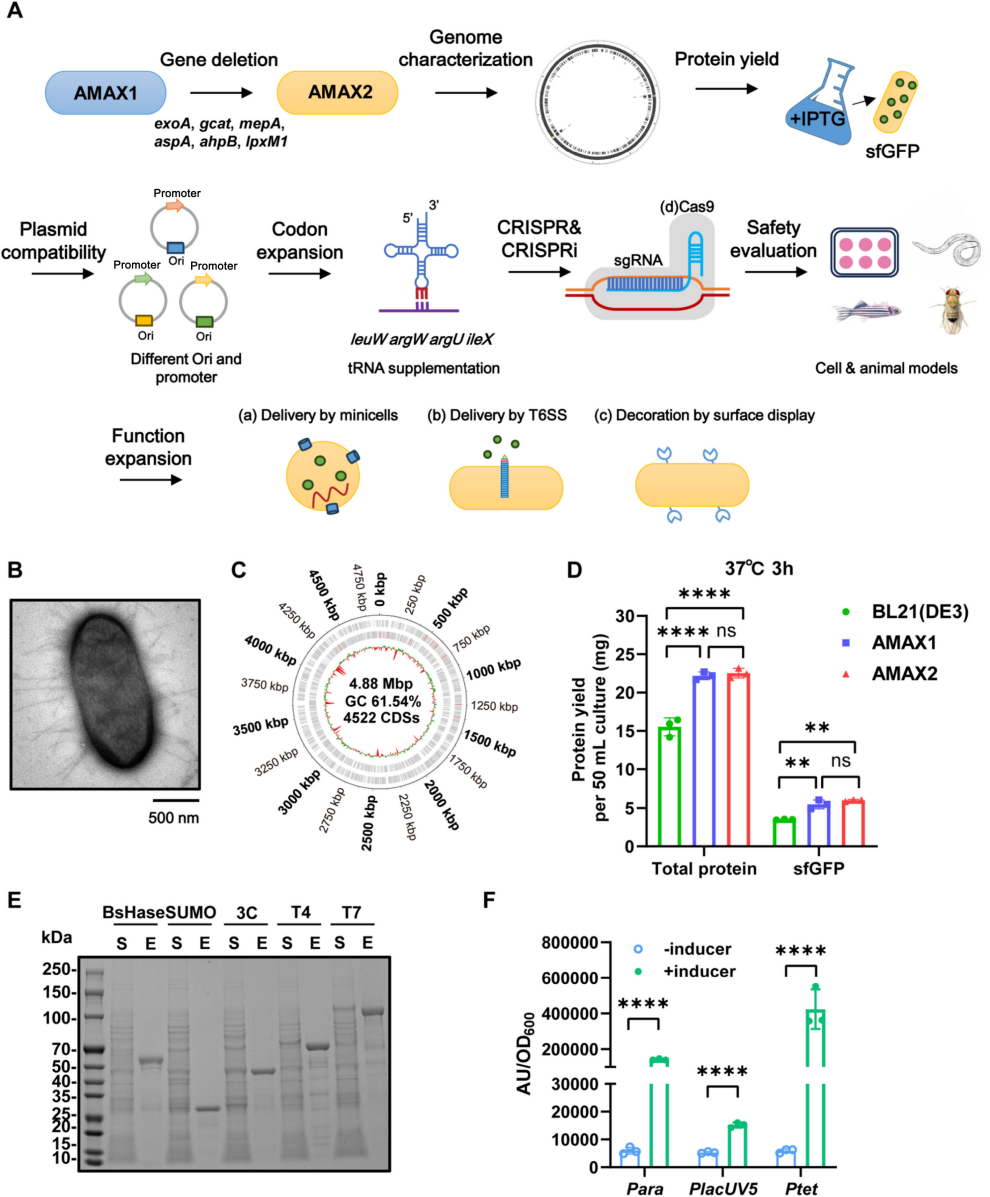
Genome features and protein expression capability of AMAX2. (**A**)AMAX2 was constructed by deleting additional virulence genes from AMAX1, followed by genome characterization and protein yield quantification to define its baseline properties. Plasmid compatibility was examined using diverse origins of replication, and protein expression was evaluated under multiple inducible systems. Codon expansion through tRNA supplementation was implemented to improve the expression of proteins enriched in rare codons, while CRISPRi was employed to screen for essential genes in AMAX2. Comprehensive *in vitro* and *in vivo* assays were performed to assess biosafety. Furthermore, AMAX2 was endowed with multifunctional capabilities, including protein delivery via minicells and contact-dependent T6SS, as well as protein surface decoration through surface display. (**B**)Transmission electron microscopy (120 kV) image of AMAX2. (**C**)Schematic representation of AMAX2 genomic features. From the outermost to the innermost circles: Genome scale: the outermost circle represents the genome’s positional scale. Genes on the + strand: the second circle depicts genes encoded on the positive (+) strand, with rRNA genes highlighted in orange. Genes on the – strand: the third circle illustrates genes encoded on the negative (–) strand. GC content: the fourth circle shows the GC content, where regions with GC content higher than the genome-wide average are marked in green, and regions with lower GC content are marked in red. (**D**)Quantification of total protein content and sfGFP yield in flask using the pET expression system in AMAX1, AMAX2, and *E. coli* BL21(DE3). The total protein yield was quantified by BCA assay, using BSA as standards. The sfGFP protein was quantified by fluorescent quantification (ex/em 480/510 nm), with purified sfGFP as the standard. (**E**)Coomassie-stained SDS-PAGE gel showing purified D-hydantoinase (BsHase), Ulp1 protease (SUMO), HRV 3C protease, T4 DNA ligase, and T7 RNA polymerase, expressed in AMAX2. S, supernatant; E, elution. (**F**)The sfGFP yield per unit biomass (AU/OD₆₀₀) in AMAX2 using different inducible expression systems. For D and F, statistical significance was determined using ordinary two-way ANOVA followed by Sidak’s for multiple comparisons. **, *P* < 0.01; ****, *P* < 0.0001; ns, not significant.

To generate and evaluate AMAX2, we established a systematic workflow including targeted gene deletions, genome characterization, and assessment of protein production and plasmid compatibility (Fig. 1A). Codon expansion, CRISPR-based genome editing and screening, and biosafety evaluation were also incorporated into this workflow to better characterize this chassis cell. In parallel, advanced modules for minicell generation, surface display, and protein secretion were included to enable specific targeting and protein delivery.

First, to further improve its biosafety profile, we deleted six additional virulence-associated genes (*exoA*, *gcat*, *mepA*, *aspA*, *ahpB*, and *lpxM1*), each implicated in toxin production, membrane modification, or host cell damage (17–19) (TableS1). The resulting strain was designated AMAX2. Transmission electron microscopy revealed that AMAX2 maintains a rod-shaped morphology decorated with multiple fimbriae (Fig. 1B). PacBio sequencing confirmed a single 4,877,836 bp circular chromosome with a GC content of 61.54%. Genome annotation using Prokka identified 4,522 protein-coding sequences, 10 copies of 23S rRNA, 11 copies of 5S rRNA, 10 copies of 16S rRNA, 127 tRNAs, and no CRISPR loci (Fig. 1C).

We next asked whether the additional deletions in AMAX2 affected its protein production capacity. Under standard conditions (0.1 mM IPTG, 37°C, 3 h), AMAX2 produced superfolder green fluorescent protein (sfGFP) at levels indistinguishable from AMAX (hereafter referred to as AMAX1) and substantially higher than *E. coli* BL21(DE3) (Fig. 1D). AMAX2 also enabled high-yield expression of five widely used enzymes, T4 DNA ligase, T7 RNA polymerase, Ulp1 protease (SUMO), HRV 3C protease, and D-hydantoinase (BsHase), which were readily purified by standard affinity purification procedures, demonstrating its suitability for industrial and laboratory-scale applications (Fig. 1E). Additionally, we show that AMAX2 also supported high-level expression from tetracycline-inducible (*Ptet*), arabinose-inducible (*Para*), and IPTG-inducible (*PlacUV5*) promoters, with sfGFP output following the order *Ptet* > *Para* > *PlacUV5* (Fig. 1F).

**Fig 2:**
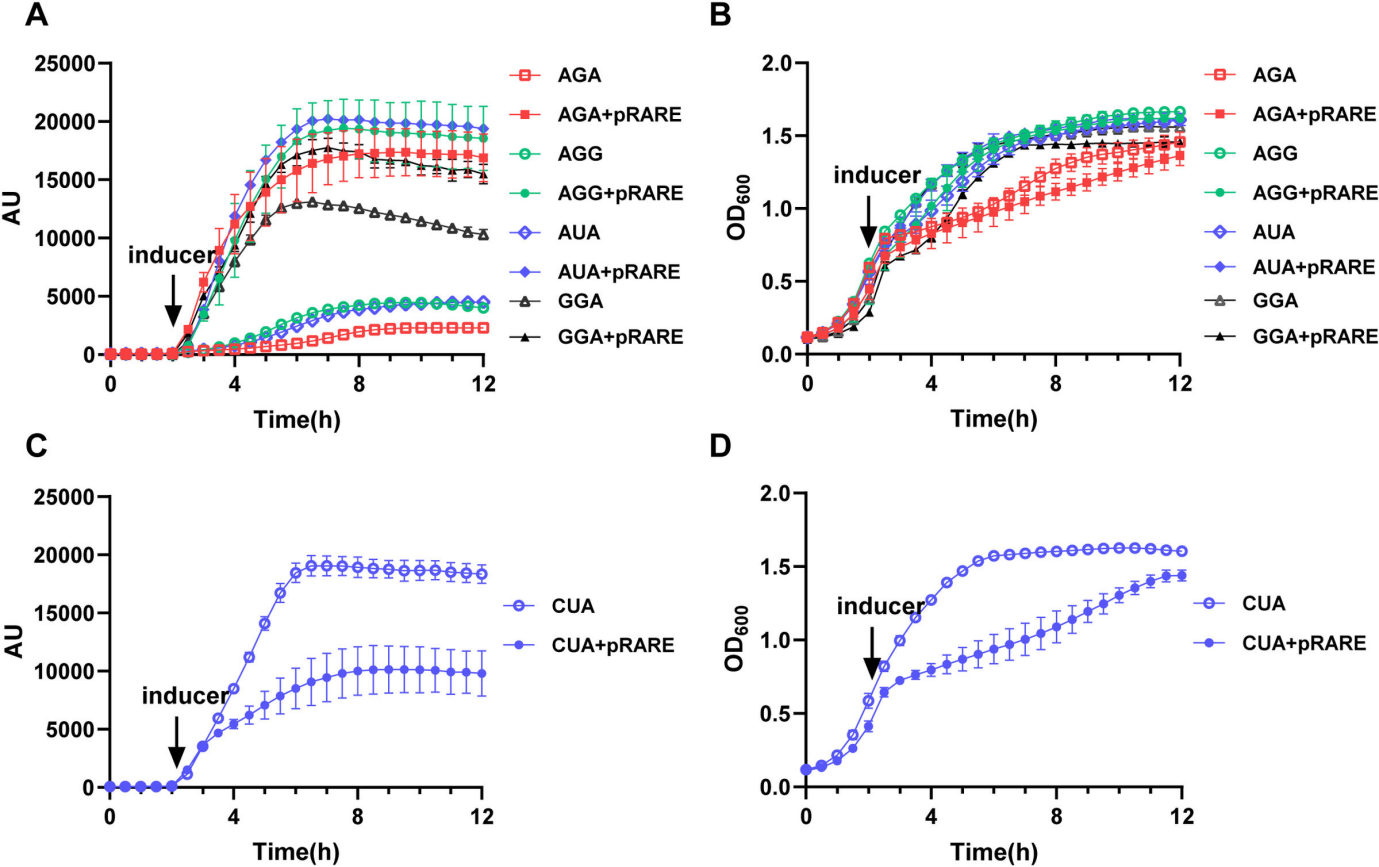
Protein expression analysis of AMAX2 harboring the pRARE plasmid. (**A and B**)The sfGFP production (**A**)and growth curves (**B**)of AMAX2 expressing the 5×AGA, AGG, AUA, and GGA-sfGFP construct with or without the pRARE plasmid under 0.1 mM IPTG induction. (**C and D**)The sfGFP yield (**C**)and growth curves (**D**)of AMAX2 expressing the 5×CUA-sfGFP construct with or without pRARE plasmid under 0.1 mM IPTG induction. For panels **A–D**, optical density at 600 nm (OD₆₀₀) and sfGFP fluorescence (ex/em 480/510 nm) were measured every 30 min using a BioTek Synergy H1 microplate reader. AU, arbitrary units.

Because rare codons can constrain heterologous expression in AMAX strains, we analyzed codon usage with CoCoPUTs (20) and identified several codons with frequencies below 5‰ (TableS2). Drawing on the *E. coli* Rosetta 2(DE3) strategy, which supplies six rare tRNAs from pRARE2 (21), we evaluated the compatibility of tRNA supplementation in AMAX2 using a panel of N-terminal 5× rare codon–sfGFP reporters. Only five of the pRARE2-encoded tRNAs (AGA, AGG, AUA, GGA, and CUA) matched the rare codons of AMAX2. Without pRARE2, sfGFP constructs containing 5×AGA, 5×AGG, or 5×AUA exhibited negligible fluorescence; co-expression with pRARE2 restored sfGFP production, confirming tRNA limitation for these codons (Fig. 2A and B). Notably, 5×GGA sfGFP already showed considerable basal expression without pRARE2 supplementation;however, its expression level was still significantly enhanced upon introduction of pRARE2 (Fig. 2A and B). Of particular interest, 5×CUA reporters expressed strongly in AMAX2, while introducing pRARE2 impaired growth and reduced sfGFP yield (Fig. 2C and D), suggesting potential incompatibility or metabolic burden from excess tRNA-Leu.

Together, these results show that AMAX2 retains the high protein expression capacity of AMAX1, supports a broad range of inducible systems, and can overcome rare codon limitations through targeted tRNA supplementation.

### CRISPR/Cas9 systems enable efficient gene editing in AMAX

**Fig 3:**
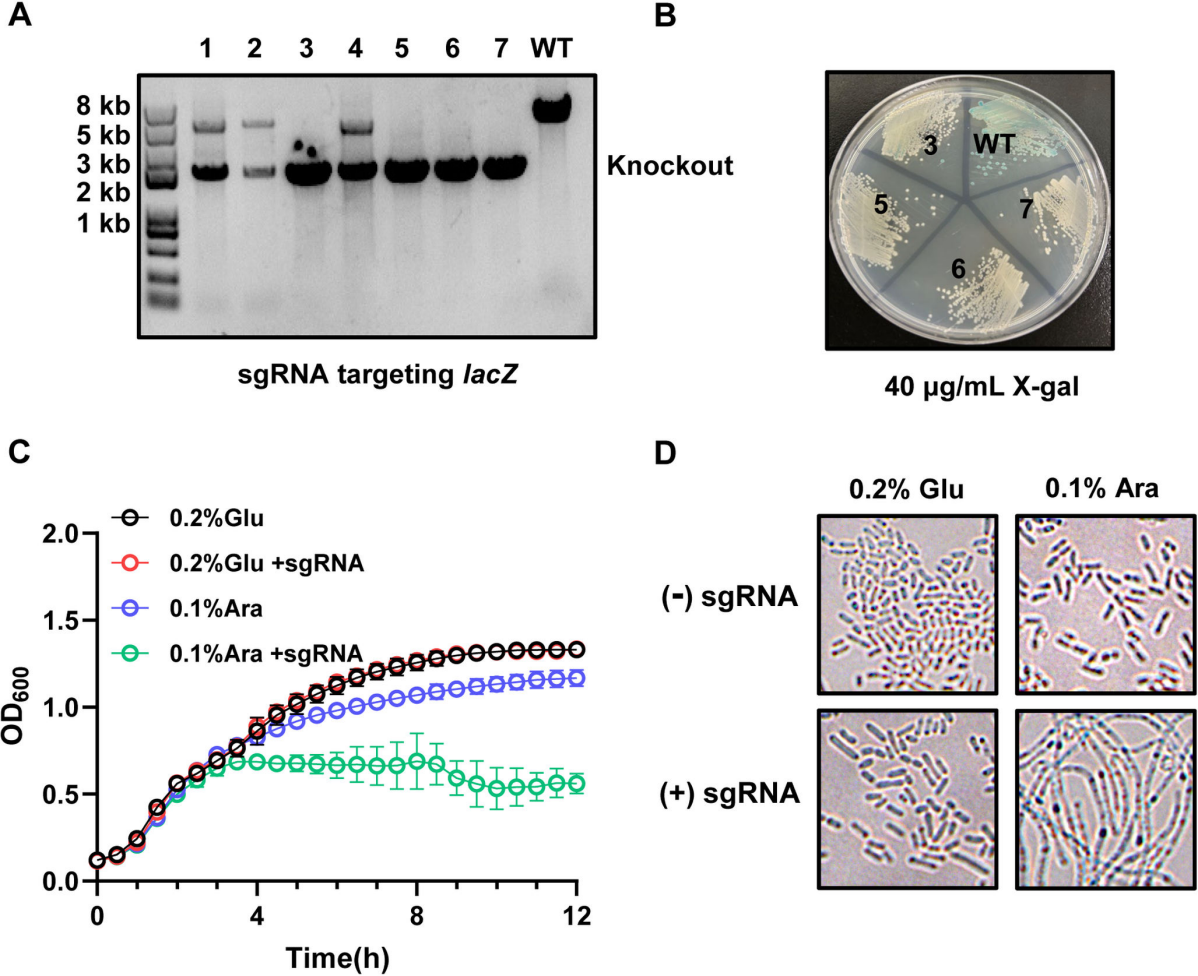
AMAX2 is compatible with the CRISPR/Cas9 system. (**A**)PCR validation of *lacZ* deletion in AMAX2 mediated by the CRISPR/Cas9 system. Primers flanking the *lacZ* target region were used to confirm successful knockout, with the expected size shift in the amplified fragment indicating deletion. (**B**)Blue-white screening on X-gal (40 μg/mL) plates verifying *lacZ* knockout. Wild-type (WT) strains with intact *lacZ* produced blue colonies, while CRISPR/Cas9-mediated knockout mutants exhibited white colonies, confirming successful gene deletion. (**C**)Growth curves of AMAX2 *Para-dCas9* strains with or without sgRNA targeting *lacZ*, supplemented with 0.1% arabinose (to activate dCas9 expression) or 0.2% glucose (to repress dCas9 expression). (**D**)Microscopy images showing cell morphology, following the induction of dCas9 and sgRNA. Ara, arabinose. Glu, glucose.

The AMAX2 strain is amenable to genetic modification via homologous recombination. To further improve its genetic tractability, we implemented the CRISPR/Cas9 system for targeted gene deletion (22, 23). The *lacZ* gene (encoding β-galactosidase) was selected as the deletion target. Knockout was performed using an sgRNA targeting *lacZ* with CRISPR/Cas9, paired with λ-Red recombination to promote double-strand break repair using a deletion construct. After antibiotic selection, seven colonies were isolated. PCR verification identified four colonies exhibiting *lacZ*-specific knockout bands (Fig. 3A). Functional validation was performed using media containing X-gal, whose cleavage by LacZ would yield a blue chromogenic product. Parental AMAX2 colonies produced a blue chromogenic product, while all four PCR-verified mutant colonies remained white, confirming the loss of enzyme function (Fig. 3B).

Additionally, we evaluated the functions of CRISPRi, which enables reversible repression of the target gene by dCas9-mediated transcriptional blocking (24–26). We first integrated an arabinose-inducible (*Para*) dCas9 cassette between *glmS* and *luxR_1* and introduced a constitutively expressed sgRNA via Tn7 transposition. The *ftsZ*, responsible for Z-ring formation during cell division, was selected as the target for function validation. We compared the growth curve and cell morphology under conditions including dCas9 expression or repression, and sgRNA presence or absence (Fig. 3C and D). The results showed that solely inducing dCas9 or expressing sgRNA alone negligibly impacted cell growth and morphology. In contrast, combined dCas9 induction and sgRNA presence significantly inhibited bacterial growth and induced elongated filamentous morphology (Fig. 3C and D). These findings confirm that CRISPRi can achieve strong and specific knockdowns in AMAX2.

Collectively, these results establish CRISPR/Cas9 systems as a robust tool for precise target gene deletion and inhibition in AMAX2.

### Genome-wide functional interrogation using pooled CRISPRi in AMAX2

To guide future genome streamlining of AMAX2, we sought to systemically identify essential and conditionally essential genes using CRISPRi. For genome-wide screening, we designed two independent sgRNAs for each annotated genetic feature using an automated selection pipeline (9). The top-ranked sgRNAs were assembled into Library Set 1 (4,586 sgRNAs), while the second-ranked formed Library Set 2 (4,667 sgRNAs). Each pool was cloned into pCRISPRi-*ccdB,* propagated in *E. coli* WM3064, and transferred into AMAX2 by optimized triparental conjugation using a helper strain harboring the Tn7 transposase plasmid. The process achieved more than 10-fold genome coverage, ensuring robust representation of all targets.

**Fig 4:**
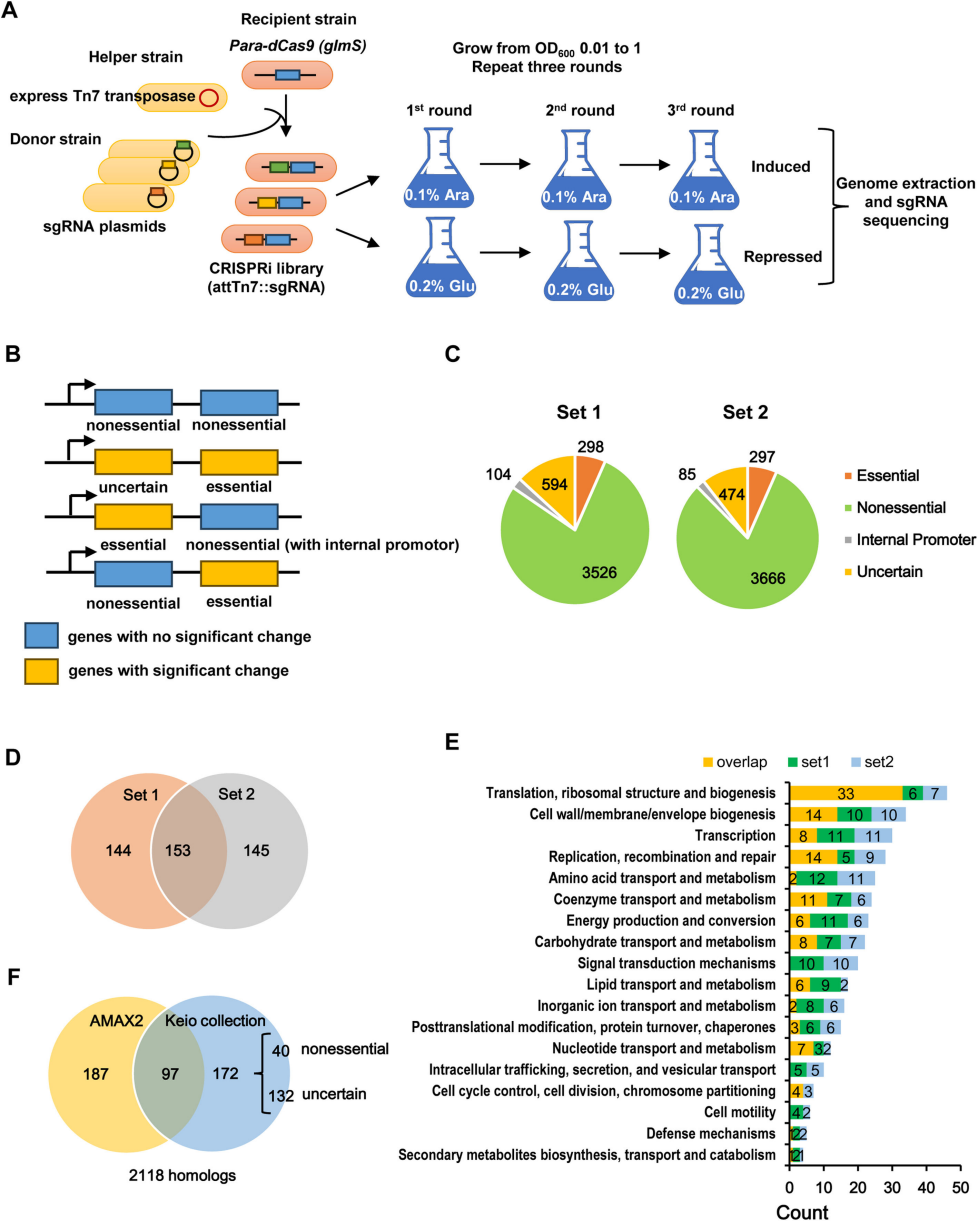
CRISPRi-seq screening reveals essential genes in AMAX2. (**A**)Schematic workflow of essential gene identification by CRISPRi-seq. The *E. coli* sgRNA library was transferred into AMAX2 *Para*-dCas9 via conjugation and integrated into the genome using the Tn7 transposase, thereby generating the sgRNA library in AMAX2. The libraries were cultured under either arabinose-induced (0.1%) or glucose-repressed (0.2%) conditions to regulate dCas9 expression. After three successive reinoculations from an initial OD₆₀₀ of 0.01–1.0, genomic DNA was extracted, and the sgRNA regions were PCR-amplified and sequenced. Comparative analysis of sgRNA abundance between the two conditions was performed, and genes with sgRNAs showing significant depletion (log₂FC < –2, adjusted *P* < 0.05) under induction were classified as candidate essential genes. (**B**)Criteria for gene classification into essential, nonessential, uncertain, and nonessential with potential internal promoters. (i) Operons with no significant change were classified as nonessential. (ii) Operons with uniform depletion were assigned as essential only for the terminal gene, while upstream genes were marked uncertain. (iii) Upstream depletion with unaffected downstream genes indicated essentiality of the upstream gene and nonessentiality of the downstream gene due to possible internal promoters. (iv) Unchanged upstream genes with depleted downstream genes were classified as nonessential (upstream) and essential (downstream).(**C**)Pie chart showing the distribution of gene categories across two datasets. (**D**)Venn diagram depicting the overlap of essential genes between Library Set 1 and Set 2. (**E**)COG functional classification of essential genes in AMAX2. (**F**)Venn diagram showing the overlap of essential genes between AMAX2 and the *E. coli* Keio collection among the 2,118 homologous genes.

We used these pooled libraries to perform genome-wide essentiality screening. Each library was grown in LB medium under either arabinose-induced (0.1%) or glucose-repressed (0.2%) conditions. Cultures were maintained in exponential phase through three consecutive 1:100 back-dilutions until reaching OD₆₀₀ 1.0. The sgRNA abundance was quantified by single-step PCR, followed by Illumina sequencing (Fig. 4A). Genes with sgRNAs showing significant depletion (log₂ fold change[FC] < –2, adjusted *P* < 0.05) under induction were classified as candidate essentials.

Given the polycistronic organization of bacterial operons, where transcriptional perturbation of upstream genes may affect downstream co-transcribed genes, we predicted operon structures in AMAX2 using OPERON-MAPPER (27). Candidate essential genes were further categorized based on operon context, as follows. (i) If all genes within an operon showed no significant change in sgRNA abundance, they were classified as nonessential genes. (ii)If all genes within an operon showed significant depletion, only the terminal gene was classified as essential, while upstream genes were designated uncertain. (iii)If the upstream gene showed significant depletion but the downstream gene did not, the upstream gene was classified as essential. The downstream gene was classified as nonessential, likely due to transcriptional compensation via an internal promoter. (iv) If the upstream gene showed no significant change but the downstream gene showed significant depletion, the upstream gene was classified as nonessential, and the downstream gene was classified as essential (Fig. 4B).

Applying these criteria, CRISPRi analysis identified 298 and 297 essential genes in Set 1 and 2 libraries, respectively (Fig. 4C). This yielded 153 shared essential genes and 289 unique essential genes distributed genome-wide (Fig. 4D). Clusters of Orthologous Groups (COG) enrichment analysis revealed their primary functions in translation, transcription, replication, cell wall/membrane/envelope biogenesis, and amino acid transport/metabolism (Fig. 4E; Fig.S1A and B). Gene Ontology (GO) enrichment analysis further demonstrated involvement in ribosome assembly and amino acid biogenesis.

We next compared AMAX2 essential genes with those in the *E. coli* Keio collection (28). Orthology analysis using SonicParanoid2 (29) identified 2,118 shared homologs between the two organisms. Of these, 97 were essential in both species, 187 were AMAX2-specific essentials, and 172 were *E. coli*–specific essentials (Fig. 4F). Among the *E. coli*-specific set, 132 homologs in AMAX2 were classified as “uncertain” due to operon positional effects despite showing depletion, while 40 were clearly nonessential. We performed Kyoto Encyclopedia of Genes and Genomes (KEGG)analysis to investigate the functions of shared and unique genes between AMAX2 and *E. coli* (30). The results showed that the genes common to both AMAX2 and *E. coli* were mainly associated with pathways including ribosome biogenesis, fatty acid synthesis, and DNA replication (Fig.S2A). In contrast, the unique gene set of AMAX2 was significantly enriched in the sulfur relay system and homologous recombination (Fig.S2B), whereas the unique genes from the *E. coli* Keio collection were mainly involved in pyrimidine metabolism and peptidoglycan biosynthesis (Fig.S2C). Notably, owing to the small number of essential genes, the enrichment *P*-values were relatively large, and no statistically significant differences were observed.

Together, our data demonstrate that AMAX2 is amenable to CRISPRi genetic screening and provide a theoretical foundation for future genome optimization.

### Generation and characterization of AMAX2 minicells

**Fig 5:**
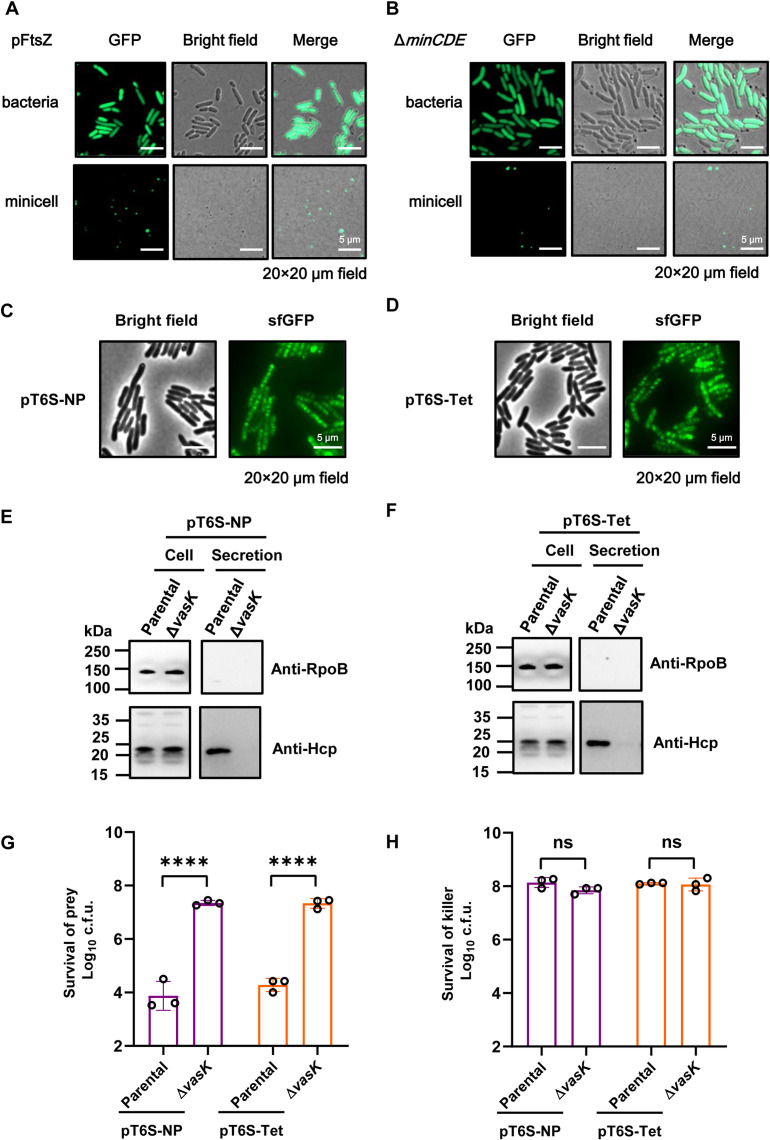
Protein delivery by minicells and T6SS. (**A**)Microscopy image of AMAX2 whole cells and minicells generated by overexpression of *ftsZ*. (**B**)Microscopy image of AMAX2 whole cells and minicells produced by the Δ*minCDE* mutant. For A and B, images were acquired using a Zeiss LSM 980 confocal microscope with Airyscan mode, and a representative 20 × 20µm field is shown. (**C and D**)Microscopy image of AMAX2 harboring pT6S-NP (**C**)and pT6S-Tet (**D**),showing localization of VipA-sfGFP. The images were captured using the Nikon Ti2-E inverted microscope,and a representative 20 × 20µm field is shown. (**E and F**)Hcp secretion assay in AMAX2 carrying pT6S-NP (**E**)or pT6S-Tet (**F**).For pT6S-Tet strains, 10 ng/mL anhydrotetracycline was added to induce expression. T6SS-deficient controls included *vasK*-deletion strains (pT6S-NP-Δ*vasK* and pT6S-Tet-Δ*vasK*). RpoB was used as a loading and intracellular protein control. Full uncropped images are presented in Fig.S4. (**G and H**)Survival of *E. coli* (**G**)and AMAX2 (**H**)after 3-h competition on LB agar plates. For pT6S-Tet strains, 10 ng/mL anhydrotetracycline was added to induce expression. Statistical significance was determined using two-way ANOVA, followed by Sidak’s multiple comparison test. ****, *P* < 0.0001; ns, not significant.

Minicells are an attractive platform for vaccine and antigen delivery, due to their ability to package proteins while lacking chromosomal DNA. To enable minicell production in AMAX2, we implemented two complementary strategies: overexpression of *ftsZ* to drive ectopic Z-ring formation at the cell poles, and deletion of the *minCDE* locus to abolish spatial control of division site positioning (31–33). Both approaches, combined with a plasmid expressing sfGFP, yielded abundant minicells that successfully recruited cytoplasmic proteins (Fig. 5A and B). Minicells generated from the Δ*minCDE* strain were further examined by cryo-electron tomography (cryo-ET). Imaging analysis revealed intact spherical particles with outer membrane diameters averaging 425.3 ± 62.5nm (range: 300–500 nm) and distinct inner membranes measuring 39.1 ± 5.1nm (Fig.S3).

### Arming AMAX2 with contact-dependent protein delivery

To extend the functional versatility of AMAX2 toward contact-dependent protein delivery, we equipped the chassis with the type VI secretion system (T6SS) from *Aeromonas dhakensis* SSU, which has been engineered onto plasmid vectors under its native constitutive promoter (pT6S-NP) or under an anhydrotetracycline-inducible promoter (pT6S-Tet) (34).

Time-lapse microscopy of strains carrying pT6S-NP or pT6S-Tet demonstrated successful T6SS expression and assembly in AMAX2, as visualized by labeling the T6SS sheath component VipA with sfGFP (Fig. 5C and D;Movie S1 and S2). Secretion assays further confirmed that AMAX2 strains with pT6S-NP or pT6S-Tet secreted the inner tube protein Hcp into the supernatant, whereas pT6S-NP-Δ*vasK* and pT6S-Tet-Δ*vasK* failed to secrete Hcp due to the loss of VasK (TssM), an essential membrane complex component (Fig. 5E and F; Fig.S4). Finally, bacterial competition assays between AMAX2 and *E. coli* revealed that AMAX2 with pT6S plasmids killed *E. coli* via T6SS-mediated effector delivery (Fig. 5G and H).

### Surface display enables specific interaction with proteins and target cells

**Fig 6:**
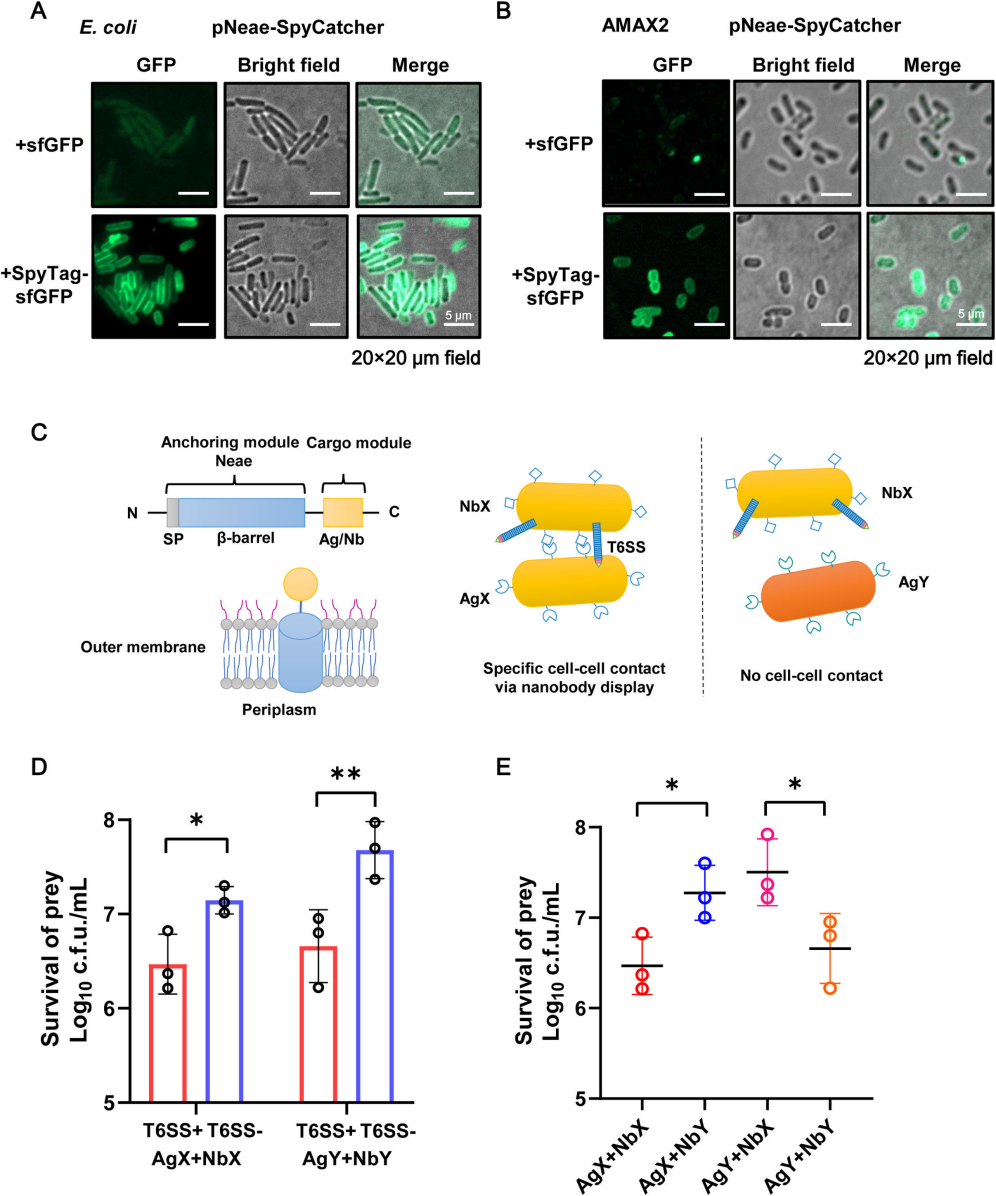
Surface engineering enables specific interaction with proteins and target cells. (**A and B**)Fluorescence image showing labeling of *E. coli* (**A**)and AMAX2 (**B**)expressing Neae-SpyCatcher with purified SpyTag-sfGFP. Free sfGFP was included as a negative control. The images were acquired using a Zeiss LSM 980 confocal microscope with Airyscan mode, and a representative 20 × 20µm field is shown. (**C**)Schematic illustrating the targeted depletion of *E. coli* by AMAX2 through specific antigen–nanobody interactions. The antigen and nanobody were displayed on the bacterial surface using the Neae anchoring module. In mixed culture, only cognate antigen-nanobody pairs mediated the binding between AMAX2 and *E. coli*, enabling AMAX2 to kill *E. coli* through T6SS-mediated contact-dependent activity. SP, signal peptide. (**D**)Survival of *E. coli* expressing Ag competed with AMAX2 harboring pT6S-NP(T6SS) or pT6S-NP-Δv*asK* (T6SS-) expressing cognate Nb following 8-hour competition in liquid LB coculture. (**E**)Survival of *E. coli* expressing Ag competed with AMAX2 harboring pT6S-NP expressing cognate or non-cognate Nb following 8-h competition in liquid LB coculture. For D–E, statistical significance was determined using two-way ANOVA, followed by Sidak’s multiple comparison test (**D**)or a two-tailed Student’s *t*-test (**E**).*, *P* < 0.05; **, *P* < 0.01.

To establish a surface display platform in AMAX2, we employed the Neae scaffold (Intimin N-terminal residues 1–659), previously validated for efficient protein presentation in *E. coli* (35, 36). To evaluate its functionality in AMAX2, we fused Neae to SpyCatcher (37) and assessed their surface localization using fluorescence-binding assays using purified proteins sfGFP and SpyTag-sfGFP (Fig.S5A and B). In both *E. coli* and AMAX2, Neae-SpyCatcher-displaying strains showed exclusive labeling with SpyTag-sfGFP, but not with sfGFP (Fig. 6A and B). These results indicate that SpyCatcher was effectively displayed on the cell surface.

Next, we tested whether AMAX2 could target specific cells by surface-displaying nanobodies (Nb) against cognate antigens (Ag). Because the T6SS delivers effectors through direct cell-cell contact, we designed competition assays using two validated nanobody-antigen pairs (AgX-NbX and AgY-NbY) (Fig. 6C) (12, 38). First, we confirmed that AMAX2 expressing AgX or AgY could employ its T6SS to eliminate target *E. coli* cells presenting the cognate nanobody NbX or NbY in liquid co-culture (Fig. 6D; Fig.S5C). To further evaluate the specificity of this T6SS-mediated killing, we performed competition assays in which AMAX2 strains harboring an active T6SS and displaying either NbX or NbY were co-cultured with target cells displaying either the cognate or a non-cognate antigen. The results revealed strict antigen-nanobody specificity: AMAX2 displaying NbX significantly reduced the survival of *E. coli* expressing AgX, but not those expressing AgY. Conversely, AMAX2 displaying NbY selectively killed targets expressing AgY, while sparing those expressing AgX (Fig. 6E; Fig.S5D).

Together, these results demonstrate that AMAX2 can be equipped with additional modules for surface specificity engineering and targeted protein delivery.

### AMAX2 displays enhanced biosafety

**Fig 7:**
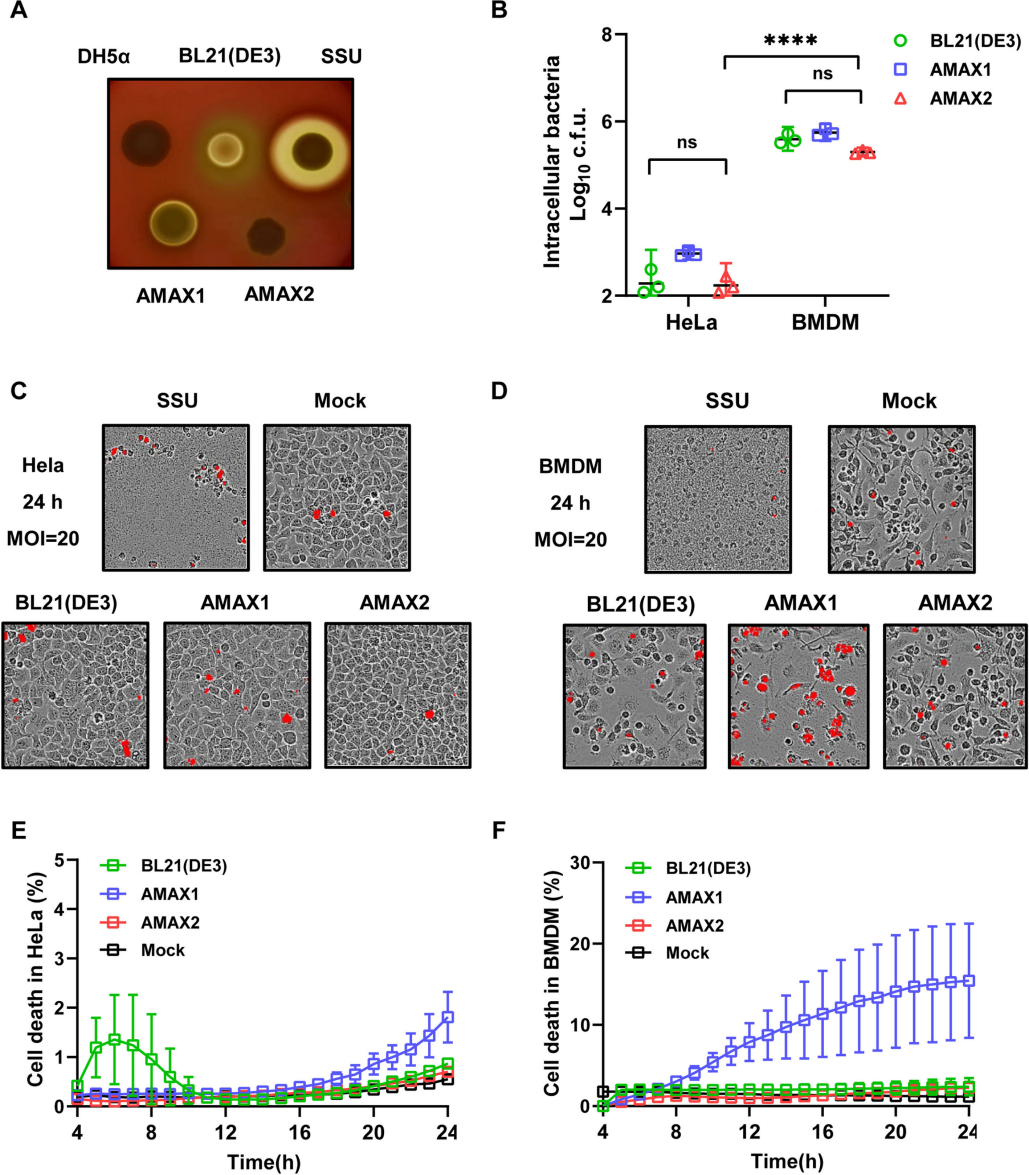
AMAX2 demonstrating comparable biosafety with BL21(DE3) in *in vitro* models. (**A**)Hemolysis assay of AMAX1, AMAX2, and BL21(DE3) on Columbia blood agar plates. *E. coli* DH5α was used as the biosafe control, and *A. dhakensis* SSU as the pathogenic control. (**B**)Intracellular bacterial load at 2 h post-infection with MOI = 20in HeLa and BMDM cells. Statistical significance was determined using two-way ANOVA, followed by a paired *t*-test for multiple comparisons. ****, *P* < 0.0001; ns, not significant. (**C and D**)Microscopy images of HeLa (**C**)and BMDM (**D**)cells 24 h post-infection, stained with PI to visualize dead cells. (**E and F**)Percentage of PI-positive HeLa (**E**)and BMDM (**F**)cells after infection with bacteria at MOI = 20. HeLa and BMDM cells were seeded in 48-well plates at a density of 2.5 × 10⁵ cells per well and infected with bacteria at an MOI of 20 for 2 h. After infection, cells were washed and treated with gentamicin and penicillin-streptomycin (PS) to eliminate extracellular bacteria. PI was then added to the medium, and cell viability was monitored hourly for 24 h using the IncuCyte imaging system.

To rigorously assess the biosafety profile of AMAX2, we benchmarked it against the laboratory reference *E. coli* BL21(DE3) and DH5α, the parental AMAX1 strain, and the opportunistic pathogen *A. dhakensis* SSU (39, 40). On Columbia sheep blood agar, AMAX2 exhibited no detectable lysis zones, mirroring DH5α. In contrast, AMAX1 produced faint β-hemolytic rings, BL21(DE3) displayed α-hemolysis with translucent halos, and SSU displayed pronounced β-hemolysis (Fig. 7A).

**Fig 8:**
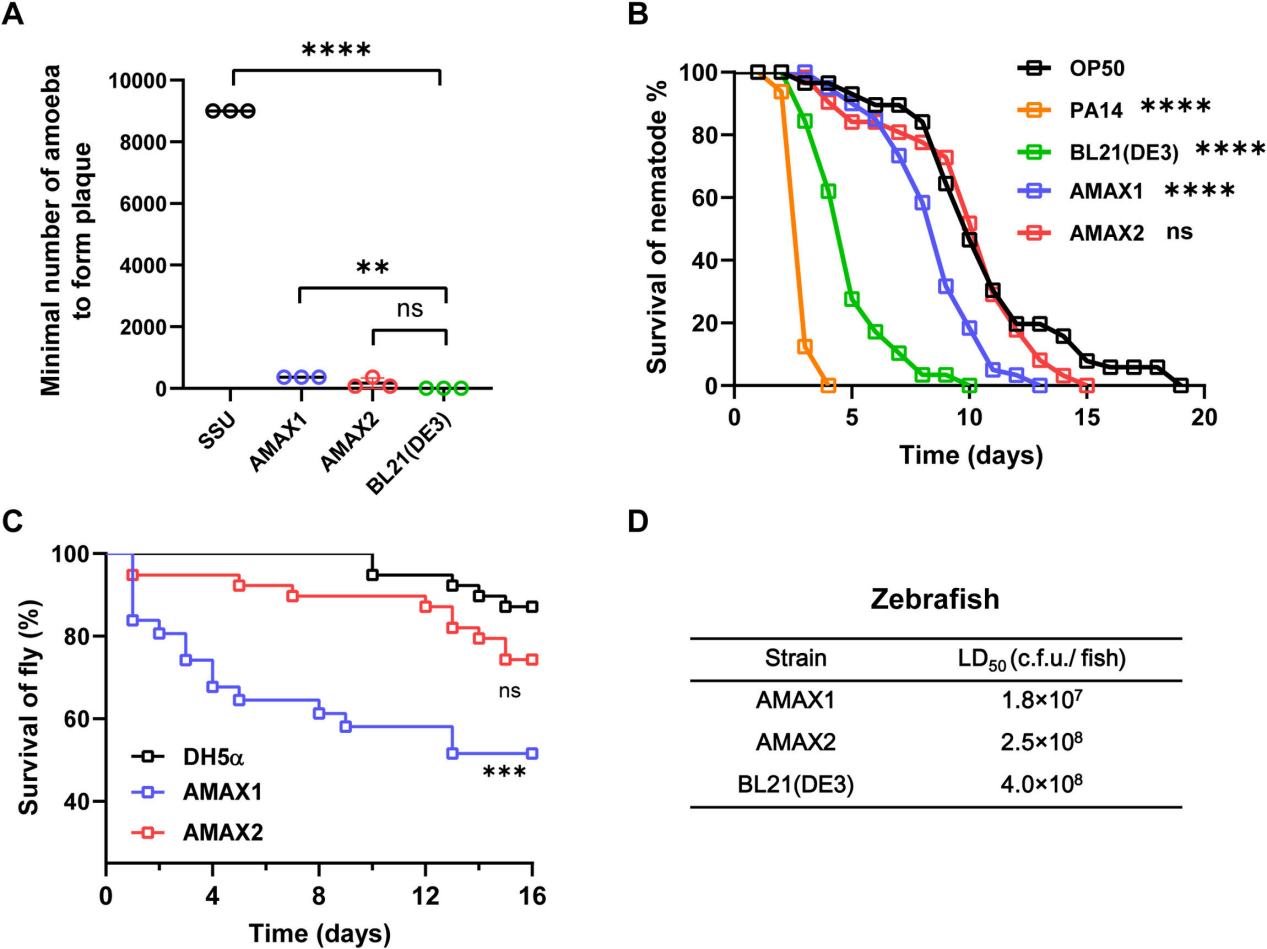
AMAX2 showing improved biosafety in *in vivo* animal models. (**A**)Phagocytic plaque formation assay using *D. discoideum*. A 5-fold serial dilution of *D. discoideum* cells (3 × 10^6^cells/mL) was spotted onto a bacterial lawn. Plates were incubated at 22°C for 3–7 days, after which plaques were counted. The lowest number of *D. discoideum* cells capable of forming a plaque on the bacterial lawn was recorded. (**B**)Survival curve of *C. elegans* following bacterial infection, with each group containing 60 nematodes. The standard food source *E. coli* OP50 and common laboratory protein expression chassis BL21(DE3) were employed as nonpathogenic controls, and the pathogenic *P. aeruginosa* PA14 served as the virulent control. (**C**)Survival curve of *D. melanogaster* following infection with different strains, with each group containing 40 male flies. The conventional laboratory strain *E. coli* DH5α was used as a nonpathogenic control. (**D**)Determination of LD₅₀ in zebrafish. Statistical significance was determined using ordinary one-way ANOVA, followed by Dunnett’s multiple comparison test (**A**),and Log-rank (Mantel-Cox) test, which evaluates overall differences across the entire observation period (**B and C**).**, *P* < 0.01; ***, *P* < 0.001; ****, *P* < 0.0001; ns, not significant.

We next evaluated cytotoxicity toward mammalian cells using two complementary models, epithelial HeLa cells and primary bone-marrow-derived macrophages (BMDMs). Propidium iodide (PI) uptake assays revealed negligible membrane damage in HeLa cells exposed to AMAX2, equivalent to BL21(DE3) and mock controls (Fig. 7B, C, and E). In contrast, SSU caused extensive rounding and detachment, while AMAX1 induced low-level cytotoxicity. Intracellular persistence assays confirmed that AMAX2 did not invade HeLa cells, whereas BMDMs, as expected, harbored viable bacteria due to phagocytosis (Fig. 7B, D, and F). In BMDMs, AMAX2 caused minimal cell death, unlike AMAX1, which elicited cytotoxicity (Fig. 7B, D, and F). In the virulence assay against the phagocytotic amoeba model *Dictyostelium discoideum*, AMAX2 also displayed improvement from AMAX1 and exhibited no virulence as BL21(DE3), while SSU was highly virulent as expected (Fig. 8A).

Finally, we extended our biosafety evaluation to three animal models—*Caenorhabditiselegans* (nematode), *Drosophila melanogaster* (fruit fly), and *Danio rerio* (zebrafish)—representing invertebrate and vertebrate hosts with progressively increasing immune system complexity (41). The nematode lacks specialized innate immune cells (41); the fruit fly has a fully developed innate immune system (42); and zebrafish, as vertebrates, possess both innate and early adaptive immunity (43). In nematode and fly infection models, AMAX2 prolonged host survival to levels indistinguishable from *E. coli* DH5α and *E. coli* OP50, a standard feed to nematode, markedly exceeding AMAX1 (Fig. 8B and C). In zebrafish, AMAX2 achieved a median lethal dose (LD₅₀) of approximately 10⁸ c.f.u./fish, equivalent to BL21(DE3) and one log higher than AMAX1 (Fig. 8D).

Taken together, AMAX2 demonstrated a safety profile indistinguishable from or superior to laboratory *E. coli* strains and significant improvement from AMAX1.

## DISCUSSION

The development of *Vibrio natriegens* Vmax marks a significant advance in the design of non-*E*. *coli* bacterial chassis for synthetic biology (44). Its rapid growth rate and robust protein expression have positioned Vmax as a widely accepted alternative to *E. coli* for recombinant protein production and other bioengineering tasks. However, its relatively low GC content (45%) (45) limits its suitability for expressing heterologous proteins derived from high-GC organisms. We recently engineered AMAX1 (GC content: 61.5%), which matches Vmax in growth kinetics while achieving significantly higher recombinant protein yields than both *E. coli* BL21(DE3) and Vmax within 3 h of induction (7). Building upon this platform, we further optimized AMAX2 with additional genetic modifications to enhance biosafety without compromising performance. Through comprehensive *in vitro* and *in vivo* safety testing and functional validation across multiple applications, we demonstrate that AMAX2 can match or exceed laboratory *E. coli* strains in safety while offering a broader functional range. This work not only establishes a set of stringent safety test benchmarks but also demonstrates diverse functions of AMAX2 as a versatile and programmable chassis platform.

Beyond safety, AMAX2 displays compatibility with diverse plasmid backbones, regulatory systems, and CRISPRi targeting. We demonstrate the utility of AMAX2 in advanced applications such as surface display with two independent systems, SpyCatcher and nanobody, for specific target interaction, and in minicell production for therapeutic delivery. Minicells, with their intrinsic inability to replicate yet retention of biosynthetic machinery, offer a unique platform for delivering proteins, nucleic acids, or small molecules to specific targets (46–48). Surface display, coupled with T6SS delivery, further extends this versatility, enabling AMAX2 to deliver proteins into specific target cells, as demonstrated by the interspecies competition assay.

Nonetheless, several issues and opportunities remain for further refinement. The AMAX2 genome sequenced with PacBio technology contains significantly fewer genes than AMAX1 genome sequenced with Nanopore technology. The reduction is likely attributable to the lower sequencing accuracy of the Nanopore platform, leading to an inflated gene count. Specifically, the gene length distributions illustrate such an annotation bias: AMAX1 contains 5,833 CDS, with 1,097 genes shorter than 300 bp and 196 shorter than 150 bp, whereas AMAX2 has 4,522 CDS, with 361 genes below 300 bp and 51 below 150 bp. In addition, the metabolic potential of AMAX, particularly in terms of value-added metabolite fermentation and stress tolerance, remains underexplored and could unlock new applications. Furthermore, although we demonstrated targeted protein delivery in prokaryotic systems, robust delivery to eukaryotic cells via minicells or protein secretion systems also warrants future efforts. Integrating orthogonal genetic or auxotrophic circuits and additional fail-safes could also enhance control in complex, open-environment applications.

Development of AMAX2 aligns well with the evolving view in how we think about microbial chassis. The future of chassis will likely not be dominated by a single or few “universal” hosts, but by a curated portfolio of specialist organisms, each with unique physiological advantages, engineered safety nets, and plug-and-play compatibility with synthetic pathways. We have previously demonstrated that AMAX is fully compatible with *E. coli* BL21(DE3) in a co-culturing system, exhibiting resistance against common *E. coli* phage contamination (7). The versatility, functional capacity, and compatibility with existing platforms highlight the potential of AMAX2 as an important chassis for biotechnological applications.

## MATERIALS AND METHODS

### Strains and growth conditions

Strains, plasmids, and primers used in this study are described in TablesS3 to S5, respectively. All constructs were verified by sequencing. Unless stated otherwise, all strains employed in the study were cultivated aerobically at 37°C with agitation at 200 rpm in LB medium ([wt/vol] 1% tryptone, 0.5% yeast extract, 0.5% NaCl). Antibiotics and inducers were used at the following concentrations: kanamycin (50 µg/mL), ampicillin (100 µg/mL), carbenicillin (100 µg/mL), chloramphenicol (25 µg/mL for *E. coli*, 2.5 µg/mL for AMAX), streptomycin (100 µg/mL), apramycin (50 µg/mL for *E. coli*, 100 µg/mL for AMAX), L-arabinose ([wt/vol] 0.1% or 0.01% as indicated), D-glucose (0.2%), IPTG (0.1 mM or 1 mM as indicated), and anhydrotetracycline (1, 10, or 100 ng/mL as indicated).

### Quantification of total protein and sfGFP yield in flask

Overnight strains were freshly inoculated and grown to OD_600_0.6 ~ 0.8 in LB media with appropriate antibiotics. Then, 0.1 mM IPTG was added for protein induction at 37°C for 3 h. After induction, cells were harvested at 6,000 × *g* for 20 min. For every 50 mL initial culture, 5 mL phosphate-buffered saline (PBS) was added to resuspend the cell pellet. The resuspension underwent sonication with 240 W for 15–20 min. Then the supernatant was collected after two-times centrifugation at 12,000 × *g* for 20 min. Total protein of each sample was quantified by Modified bicinchoninic acid (BCA) Protein Assay Kit (NO. C503051, Sangon Biotech) with absorbance at 562 nm, using bovine serum albumin (BSA) as standards. The sfGFP protein was quantified by fluorescent quantification (ex/em 480/510 nm), with purified sfGFP as the standard. The concentration of the sfGFP standard was also determined by the BCA assay.

### Determination of growth and sfGFP yield by microplate reader

Overnight cultures were sub-inoculated into black-walled, clear-bottom 96-well sterile plates, with each well containing 150 μL of culture. After incubation at 37°C for 2 h to facilitate growth, inducers were added to each well to initiate protein expression. Optical density at 600 nm (OD₆₀₀) and sfGFP fluorescence (ex/em 480/510 nm) were measured every 30 min using a BioTek Synergy H1 microplate reader.

### Protein purification using AMAX2

Overnight cultures were sub-inoculated into 200 mL of fresh LB medium supplemented with the appropriate antibiotics. When the cell density reached an OD_600_ of~0.6, protein expression was induced by adding 0.5 mM IPTG, followed by incubation at 37°C for 3 h. Cells were then harvested by centrifugation at 5,000 × *g* for 15 min.

For His-tagged proteins, the cell pellets were resuspended in lysis buffer (50 mM NaH_2_PO_4_, 300 mM NaCl, 5% glycerol, 20 mM imidazole, pH 8.0) and lysed using high-pressure homogenization. The lysate was clarified by centrifugation at 15,000 × *g* for 40 min, and the supernatant was incubated with Nickel-Nitrilotriacetic Acid (NTA) Agarose Beads for affinity purification. After washing, bound proteins were eluted using an imidazole gradient (50–400 mM) in the same buffer.

For GST-tagged proteins, the cell pellets were resuspended in lysis buffer (50 mM NaH_2_PO_4_, 300 mM NaCl, 5% glycerol, pH 8.0) and lysed. Following centrifugation, the supernatant was incubated with Glutathione Agarose Beads to capture GST-fusion proteins. The target proteins were eluted with a reduced glutathione gradient (5–20 mM).

### 120 kV transmission microscopy

Samples were immobilized on 400-mesh copper grids for 1 min, followed by negative staining with 3% uranyl acetate for 30 s. Grids were air-dried under ambient conditions prior to imaging. TEM was performed using a Thermo Scientific Talos 120C transmission electron microscope.

### Genome sequencing and annotation of AMAX2

Genome sequencing services were provided by Guangdong Magigene Biotechnology Co., Ltd. (Guangzhou, China). Genomic DNA was extracted using a commercial DNA extraction kit following the manufacturer’s protocol. DNA integrity and purity were assessed by 1% agarose gel electrophoresis, while concentration and purity were quantified using a Qubit 3.0 Fluorometer and NanoDrop One spectrophotometer (Thermo Fisher Scientific, Waltham, MA, USA). Qualified DNA was fragmented using G-tubes, subjected to end repair, and processed into SMRTbell template libraries per the manufacturer’s guidelines. Library quality was assessed with a Qubit 4.0 Fluorometer (Life Technologies, Grand Island, NY, USA), and the average fragment size was determined using an Agilent 4200 TapeStation system (Agilent Technologies, Santa Clara, CA, USA). SMRT sequencing was performed on a PacBio Revio instrument (Pacific Biosciences, Menlo Park, CA, USA) using standard protocols.

Raw reads underwent quality control processing, including removal of low-quality sequences. The resulting high-quality data (average coverage depth: 245.5×; genome coverage: 100%) were used for *de novo* assembly with Flye v2.9.2-b178. Genome annotation was performed using Prokka (v1.13), and functional annotation of the predicted proteome was conducted with eggNOG-mapper (v2.1.12), assigning Gene Ontology (GO) terms, Kyoto Encyclopedia of Genes and Genomes (KEGG) pathway annotations, and Clusters of Orthologous Groups (COG) categories.

### CRISPR/Cas9 knockout

The CRISPR knockout procedure was performed according to established protocols (23). Briefly, the pCas9 plasmid was introduced into AMAX cells via conjugation with selection on LB plates with 10 μg/mL tetracycline. Positive colonies were inoculated and grown in fresh LB medium to an OD₆₀₀ of~0.6. Cells were induced with 0.1% arabinose for 2 h to express Cas9 and the λ-Red recombinase system. After induction, cells were harvested and rendered electrocompetent. The pCRISPR plasmid (encoding sgRNA and homology repair arms) was electroporated into the cells, and transformants were selected using 10 μg/mL tetracycline and 100 μg/mL carbenicillin. A successful knockout was confirmed by PCR analysis.

To cure plasmids post-editing, the PCR-verified knockout strain was grown in LB broth and plated on LB-no salt (LBNS, [wt/vol] 1% tryptone, 0.5% yeast extract) agar plates containing 5% sucrose. Individual sucrose-resistant colonies were randomly picked and streaked onto antibiotic-free, tetracycline, and carbenicillin LB plates, respectively. Plasmid cure was confirmed by observing growth only on antibiotic-free plates, with no growth on tetracycline or carbenicillin plates, indicating elimination of both pCas9 and pCRISPR plasmids.

### Construction of CRISPRi library

Briefly, the *E. coli* sgRNA library was constructed as previously described (9). The sgRNAs were designed using a previously published R script for automated sgRNA selection (https://github.com/veeninglab/CRISPRi-seq). Two sgRNAs were designed per gene and assigned to two sets: the highest-ranking sgRNA (based on the selection script criteria) comprised Set 1, and the second-highest comprised Set 2. For genes with fewer than two available PAM sites in their coding regions, only one sgRNA was designed for Set 1. Each oligo contained the specific 20-nt sgRNA spacer flanked by two BsaI sites, a set-specific sequence (for amplifying either Set 1 or Set 2 sgRNAs), and a universal sequence for amplifying all sgRNAs on the chip.

The pooled sgRNA oligos for Set 1 and Set 2 were synthesized as a single oligo chip by GenScript (Nanjing, China) and amplified using a universal primer. The amplicons were purified by Monarch PCR & DNA Cleanup Kit (5 µg) (#T1030, NEB) and cloned into the plasmid pCRISPRi-*ccdB* via BsaI-mediated Golden Gate Assembly. Each 20-µL Golden Gate Assembly reaction product was transformed into chemically competent *E. coli* WM3064; successful transformants were selected on LB agar plates containing 50 µg/mL apramycin and 100 µg/mL 2-6-diaminopimelic acid (DAP). Twenty-five independent 20-µL reactions were performed and transformed. For each sgRNA pool, over 50,000 transformant colonies were harvested, representing more than 10-fold coverage of the sgRNA library.

The *E. coli* WM3064 donor strains harboring the sgRNA library were conjugated into the AMAX2 *Para-dCas9* recipient strain. Conjugation employed a helper WM3064 strain harboring plasmid pJMP1039, which expresses TnsABCD transposase. Successful transconjugants, which contained the Tn7 cassette integrated into the chromosome, were selected on LB agar plates supplemented with 100 µg/mL apramycin at 37°C. Approximately 50,000 colonies were collected for each set (Set 1 and Set 2) sgRNA pool during the final library construction step.

### CRISPRi-seq screening

The pooled libraries were sub-cultured in LB broth at 37°C for 3 h. Subsequently, the libraries were inoculated into fresh LB medium supplemented with either 0.2% glucose or 0.1% arabinose, starting at an initial OD_600_ of~0.01. After approximately 2.5 h of growth (reaching OD_600_~1), the cultures were re-diluted into fresh medium containing the same inducer to an OD_600_ of~0.01. This serial dilution cycle was repeated three times. Following the final dilution cycle, libraries grown in LB with 0.2% glucose or 0.1% arabinose were harvested. Genomic DNA was extracted using the TIANamp Bacterial DNA Kit (#DP302, TIANGEN) according to the manufacturer’s protocol. The sgRNA regions were then amplified from the genomic DNA using specific primers, and the resulting amplicons were submitted for Illumina sequencing.

### Data analysis

The absolute abundance of each sgRNA per condition was robustly determined from the raw paired-end sequencing data using 2FAST2Q (v2.5.0). Applying 2FAST2Q’s default configuration, a nucleotide-based quality filtering step retained only trimmed reads with Phred scores ≥ 30, discarding lower-quality data. Differential abundance analysis of sgRNAs was performed using DESeq2 (v1.42.1) within the R statistical environment (v4.3.3). Putative essential hits were defined as sgRNAs exhibiting a Log_2_ FC < −2 and adjusted *P* < 0.05. Operons in AMAX were predicted using the OPERON-MAPPER tool (https://biocomputo.ibt.unam.mx/operon_mapper/) (27).

For gene categorization, genes were initially classified into four distinct groups: “Nonessential,”“Uncertain,”“Essential,” and “Nonessential with internal promoter,” based on established criteria (49, 50) and summarized in Fig. 3C. The predicted list of essential genes in AMAX was subsequently benchmarked against the *E. coli* Keio essential gene collection (28). Homology mapping between AMAX and *E. coli* genes was established using SonicParanoid2 (v2.0.8) (29). KEGG analysis was performed on the shared and unique essential genes in AMAX2 and *E. coli* (30). All analyzed data are provided in the supplemental material, including sgRNA abundance analysis for Set 1 and Set 2, as well as homology analysis between AMAX2 and *E. coli*.

### Collection of minicells

Overnight cultures were inoculated into 400 mL fresh LB medium and grown until reaching an OD_600_ of~0.6 (adding 0.1% arabinose to induce FtsZ overexpression if needed). The sfGFP was induced with 0.1 mM IPTG by 3 h at 37°C. Then, the cells were pelleted by centrifugation at 6,000 × *g* for 20 min, and the supernatant was collected, filtered through a 0.8-μm membrane, and concentrated using a 100-kDa cutoff ultrafiltration device. Minicells were then isolated by ultracentrifugation (20,000 × *g*, 1 h) and resuspended in 400 μL PBS.

### 300 kV cryo-electron tomography sample preparation

Samples suspended in PBS were gently mixed with 10-nm colloidal gold particles (used as fiducial markers) immediately before plunge freezing. For cryo-EM grid preparation, 3 μL of the sample was applied onto freshly glow-discharged Quantifoil R2/1 carbon-coated copper grids (200 mesh), incubated for 5 s, and then blotted with filter paper. After a blotting wait time of 3.5 s, the grids were plunge-frozen in liquid ethane using a Vitrobot Mark IV system (Thermo Scientific) operated under 100% humidity and room temperature. Frozen grids were stored in liquid nitrogen until data collection.

### Cryo-ET data acquisition and tomogram reconstruction

Cryo-ET data were acquired on a Titan Krios transmission electron microscope (Thermo Fisher Scientific) operated at 300 kV, equipped with a K2 Summit direct electron detector (Gatan Inc.). Tilt series were collected using SerialEM software (51) from −60° to+60° with 3° increments using a unidirectional acquisition strategy. Images were recorded in counting mode at a nominal magnification of 81,000×, resulting in a pixel size of 12.433 Å/pixel. Each tilt image was recorded as a movie of 10 frames with an individual frame exposure time of 0.092 s, corresponding to a total exposure time of 0.92 s and a dose of~3.65 e⁻/Å² per stack. The total accumulated dose across the full tilt series was approximately 150 e⁻/Å². The energy filter slit width was set to 20 eV, and the defocus range was set from −2.5 μm to −5.5 μm. Beam-induced motion correction was performed using MotionCor2 (52). Tilt series alignment was performed in IMOD (53) using bead-tracking, and tomograms were reconstructed using the weighted back-projection method implemented in the same software.

### Time-lapse fluorescence microscopy

Strains harboring pT6S-NP plasmids were cultured in LB broth with appropriate antibiotics to OD_600_ ~ 1. Strains carrying pT6S-Tet plasmids were grown to OD_600_ ~ 0.6, induced with 100 ng/mL anhydrotetracycline (aTc) at 37°C for 30 min, then centrifuged at 4,500 × *g*for 2 min. Cell pellets were resuspended in fresh LB broth (supplemented with aTc when required) and spotted onto 1% agarose pads prepared with 1 × LB medium. Imaging was performed at 37°C using a Nikon Ti2-E inverted microscope equipped with a 100×/1.45 NA oil-immersion objective and ET-GFP filter set (Chroma #49002). Images were captured with a duration of 5 min with an interval of 10 s. All experiments were conducted in two independent biological replicates, with representative images shown.

### Protein secretion assay

Overnight cultures were inoculated into fresh LB medium with appropriate antibiotics inducers as indicated and grown to OD_600_  ~ 1at 30°C. Then, 2 mL of OD_600_ ~ 1cells was collected by centrifugation at 6,000  ×  *g*for 2 min and resuspended in 1 mL fresh LB. Resuspended cells were placed at 30°C for 3 h and centrifuged at 10,000  ×  *g*for 2 min at room temperature. The cell pellet was used as the whole-cell sample. The supernatant was centrifuged at 10,000  ×  *g*for 2 min again as the secretion sample. All samples were mixed with SDS-loading dye, boiled at 98°C for 10 min, followed by sodium dodecyl sulfate-polyacrylamide gel electrophoresis (SDS-PAGE) analysis and western blotting analysis.

### Western blotting analysis

After electrophoresis in a 12% SDS-PAGE gel, proteins were transferred to a PVDF membrane (Bio-Rad). Then, the protein-bound membrane was blocked with 5%(wt/vol)non-fat milk in TBST (50 mM Tris, 150 mM NaCl, 0.1% [vol/vol] Tween-20, pH 7.6) buffer for 1 h at RT. After blocking, the membrane was sequentially incubated with primary and secondary HRP (horseradish peroxidase)-conjugated antibodies in TBST with 1%(wt/vol) milk for 1 h at RT. Signals were detected using the Clarity ECL solution (Bio-Rad). The commercially available monoclonal RpoB (Biolegend, #663905) and V5 antibody (Invitrogen, #R960CUS) were used. The polyclonal antibody to Hcp was custom-made by Shanghai Youlong Biotech. The HRP-linked secondary antibodies were purchased from ZSGB-Bio (Product # ZB-2305 [mouse] and # ZB-2301 [rabbit]).

### Bacterial competition assay

For solid plate competition assays, overnight cultures of predator and prey strains were sub-cultured in fresh LB with appropriate antibiotics to OD_600_ ~ 1 (with aTc as indicated). Competing cells were mixed at a 10:1 ratio and co-incubated on LB agar plates at 37°C for 3 h. For liquid coculture competition, overnight cultures were diluted in fresh LB supplemented with 0.1% arabinose (for Neae-Nb/Neae-Ag induction) to an initial OD_600_ of 0.03 (predator) and 0.003 (prey), maintaining a 10:1 ratio. Cocultures were shaken at 200 rpm (37°C) for 8 h or 24 h. Following co-incubation, viable cells were quantified by 10-fold serial dilution plating on selective LB agar plates. The mean Log_10_ c.f.u. of the recovered strains was plotted, and error bars show the mean ± standard deviation of at least three biological replicates. Statistical significance was determined by one-way or two-way ANOVA with Tukey’s multiple comparisons test (*P* values reported).

### SpyCatcher/SpyTag labeling assay

Overnight cultures were inoculated into fresh LB medium containing 0.1% arabinose and grown at 37°C until reaching an OD_600_ ~ 1. The cells were then pelleted and resuspended in PBS with 10 µM purified sfGFP or SpyTag-sfGFP, respectively. After overnight incubation at 4°C, the unbound protein was washed three times with PBS. The bacterial cells were then spotted on the PBS-1% agarose pad. Fluorescence imaging was conducted using a Zeiss 980 confocal microscope.

### Hemolytic assay

The hemolytic activity was determined by using Columbia agar containing 5% (wt/vol) sheep blood, and the plates were incubated at 37°C for 24–48 h. The hemolytic activity was evaluated and classified on the basis of lysis of red blood cells in the medium around the colonies: the green zones around colonies (α-hemolysis), clear zones around colonies (β-hemolysis), and no zones around colonies (γ-hemolysis) on plates. Only strains with γ-hemolysis are considered safe.

### Cell toxicity assay

The cells were seeded in 48-well plates at a density of 2.5 × 10^5^cells per well in DMEM supplemented with 10%fetal bovine serum (FBS). Bacteria at varying MOIs were introduced into the wells and incubated for 2 h. Post-infection, the cells were washed with PBS and exposed to DMEM containing 300 μg/mL gentamicin and 10% penicillin-streptomycin (PS) for 2 h to eradicate any remaining extracellular bacteria. For quantifying intracellular bacteria, the cells underwent three PBS washes before being lysed with 1% Triton X-100 at 37°C for 3 min. The lysates were then subjected to 10-fold serial dilutions on LB plates to evaluate the survival of internalized bacteria. To assess cell death post-infection, the cells were washed again following the elimination of extracellular bacteria and cultured in DMEM containing 10 μg/mL gentamicin to inhibit the growth of extracellular bacteria. Subsequently, PI (Abcam, ab14083) was added to the medium, and cell viability was monitored every hour using the IncuCyte system over a 24-h period.

### Amoeba phagocytic assay

Overnight strains were inoculated into fresh LB and grown to OD_600_ ~ 1. Cells were collected by centrifugation at 10,000 × *g*for 1 min and resuspended to OD_600_ ~ 1in 1 mL PBS. A 300-μL volume of bacterial cells was plated on SM/5 plates (2 g glucose, 2 g protease peptone, 0.2 g yeast extract, 0.2 g MgSO_4_·7H_2_O, 0.38 g KH_2_PO_4_, and 0.12 g K_2_HPO_4_/L, pH to 6.0–6.4) and allowed to dry at room temperature. *D. discoideum* cells were pelleted by centrifugation at 500 × *g*for 5 min, washed once with PBS, and resuspended in PBS to a final concentration of about 3  ×  10^6^cells/mL. A series of 5-fold dilutions of *D. discoideum* cells was plotted on the SM/5 plates. The plaques were counted after plates were incubated at 22°C for 3–7 days. The minimal number of *D. discoideum* cells deposited above that was able to form plaque on the bacterial lawn was recorded.

### Fly oral infection assay

For the oral infection procedure, 8- to 10-day-old adult male flies were starved for 3 h in empty vials at 28°C to ensure synchronous feeding when transferred to the infection vials. Bacteria were grown in LB medium to the exponential phase (OD_600_ ~ 1) at 37°C and then resuspended to an OD_600_ of 40 using LB supplemented with 5% sucrose. The starved flies were then flipped into the infection vials containing standard media, which were covered with Whatman paper disks soaked in 150 µL of the bacterial solution. The flies were allowed to feed in the infection vials for 16 h, after which they were transferred to fresh food vials. Each group contains 40 flies.

### Nematode infection assay

Different bacterial strains were inoculated into LB medium and incubated overnight at 37°C. The overnight cultures were then spread evenly onto 35 mm NGM agar plates, modified to contain 0.35% peptone instead of 0.25%. These plates were incubated at 37°C for 24 h, followed by an additional incubation at 25°C for 16–24 h, before seeding with synchronized L4-stage *C. elegans*. The slow-killing assay was conducted at 25°C, with animal survival monitored every 12 h. To prevent the hatching of progeny, nematodes were transferred to fresh NGM plates daily. Each group included 60 worms, and worms were considered dead if they failed to respond to a gentle touch.

### Zebrafish LD_50_ determination

All zebrafish (90-day-old, 2–3g/fish) used in this research were derived from a single parental AB strain. Before infection, all bacterial strains were cultured in LB (37°C, 200 rpm) to OD_600_ ~ 1. The fresh bacterial culture was then diluted to different concentrations and resuspended in PBS, using OD_600_ = 1containing2 × 10^8^CFU/mL bacteria as a standard. For each strain, five gradient concentrations were prepared, with at least five fish per concentration group. Zebrafish in each group were infected via intraperitoneal injection, and the mortality rates were recorded in a week. The LD_50_ were calculated using Probit regression analysis in SPSS v29.0, all regression equations yielding *P* < 0.05.

## Data Availability

The genome and CRISPRi data for AMAX2 are available in the National Microbiology Data Center under accession numbers NMDC60214071 and NMDC10020694, respectively. Data supporting the findings of this study are available within the paper or from the corresponding author upon request.

## SUPPLEMENTAL MATERIAL

Data Set S1 aem.00231-26-s0001.xlsx CRISPRi analysis.

Supplemental material aem.00231-26-s0002.pdf Fig. S1 to S5; Tables S1 to S5.

Supplemental legends aem.00231-26-s0003.pdf Descriptive legends for Movies S1 and S2.

Movie S1 aem.00231-26-s0004.avi Time-lapse movie showing VipA-sfGFP localization in AMAX2 with pT6S-Np.

Movie S2 aem.00231-26-s0005.avi Time-lapse movie showing VipA-sfGFP localization in AMAX2 with pT6S-Tet.

ASM does not own the copyrights to Supplemental Material that may be linked to, or accessed through, an article. The authors have granted ASM a non-exclusive, world-wide license to publish the Supplemental Material files. Please contact the corresponding author directly for reuse.
