# Supplementary material for: A programmable Aeromonas chassis, AMAX2, for advanced biomanufacturing
Source: Appl Environ Microbiol. 2026 May 18;92(6):e00231-26. doi: 10.1128/aem.00231-26 (PMC13274425; doi:10.1128/aem.00231-26)
Supplement: Supplemental material — Fig. S1 to S5; Tables S1 to S5. [file aem.00231-26-s0002.pdf]

Supplementary File

A programmable *Aeromonas* chassis AMAX2 for advanced biomanufacturing

Ming-Xuan Tang, Yu-Zhao Liu, Jia-Xin Liang, Ruo-Lin Huang, Xuepiao Pu, Chen-Chen Liang, Zi-Yu Tang, Tong-Tong Pei, Ya-Jie Zhao, Hao-Yu Zheng, Tingting Zhang, Zixian Wu, Ying An, Xiaoye Liang, Xue Liu, Tao Dong

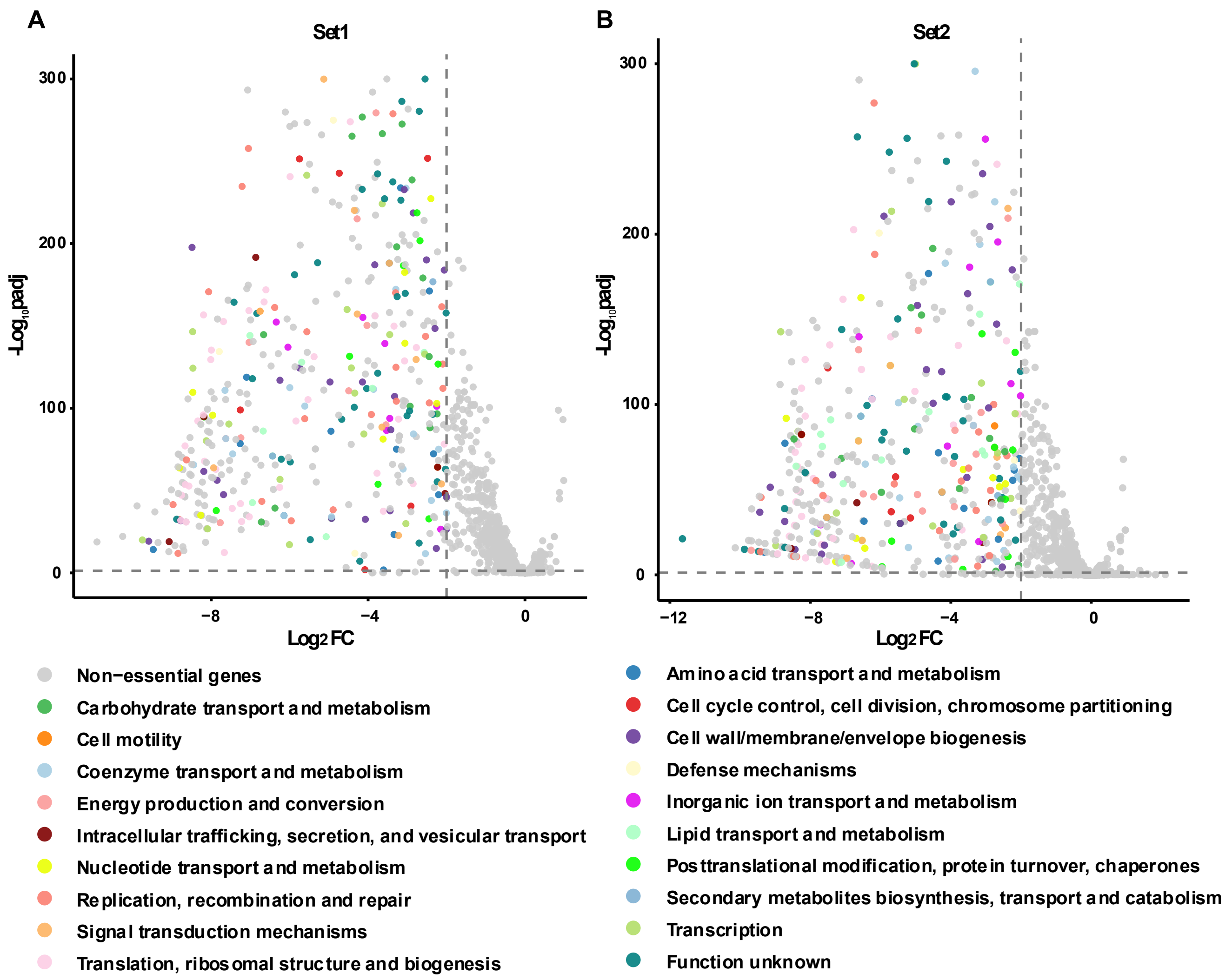

Figure S1. Volcano plots showing the COG functional classification of essential genes in Set1 (A) and Set2 (B). The cutoff for hit genes was set at $\log_2$ FC (fold change) < −2 with an adjusted *p*-value (padj) < 0.05. Genes with significant changes were defined as essential after excluding uncertain proteins resulting from polycistronic effects.

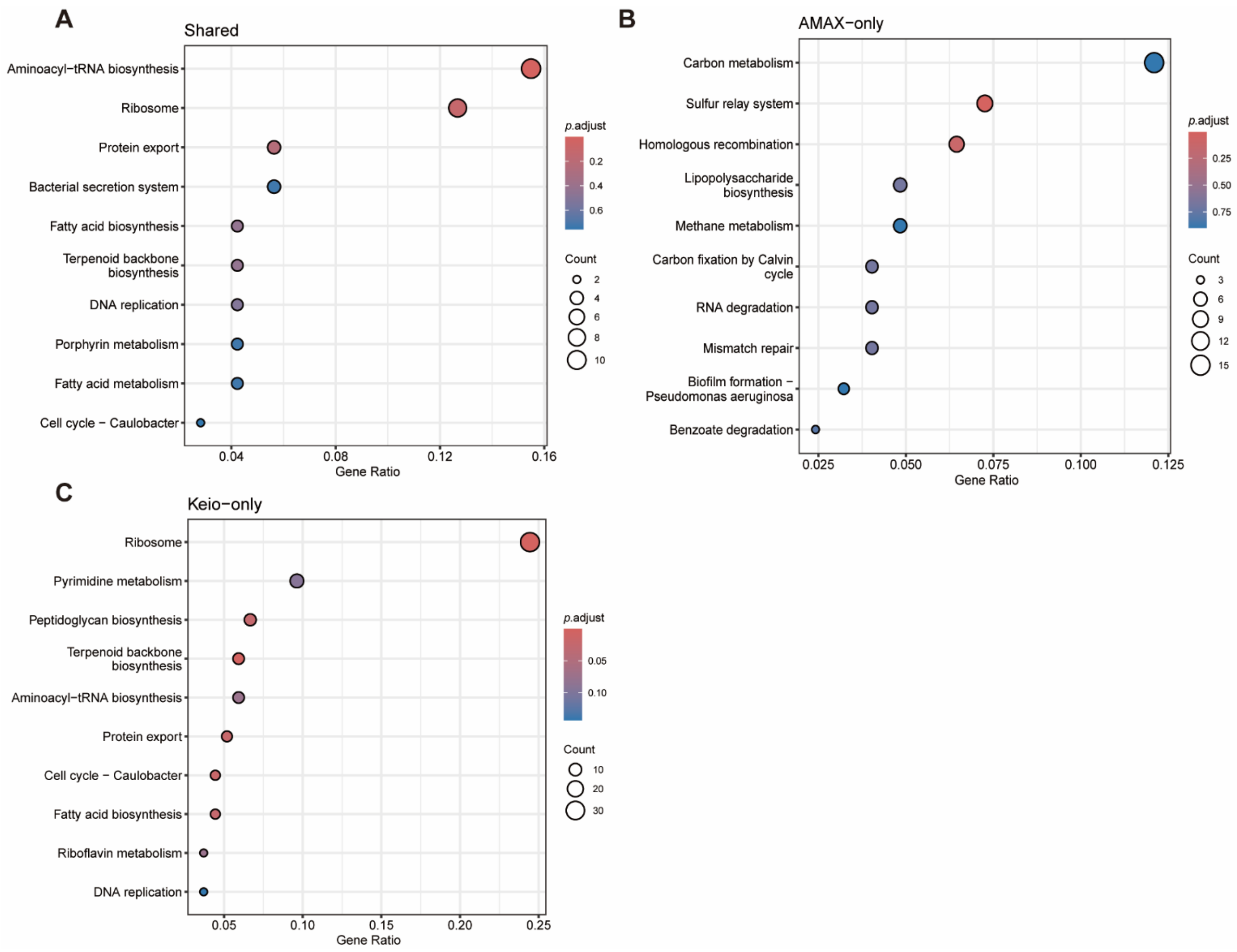

Figure S2. KEGG analysis of shared and unique essential genes from CRISPRi screening in AMAX2 and the *E. coli*. Bubble plot illustrating KEGG enrichment results for shared genes (A), AMAX2-specific genes (B), and *E. coli* Keio collection–specific essential genes (C). In each plot, the size of each bubble represents the number of enriched genes corresponding to the respective pathway, while the color gradient indicates the significance of enrichment (*p*. adjust). Gene Ratio**:** Number of enriched genes in the pathway/ total number of input genes.

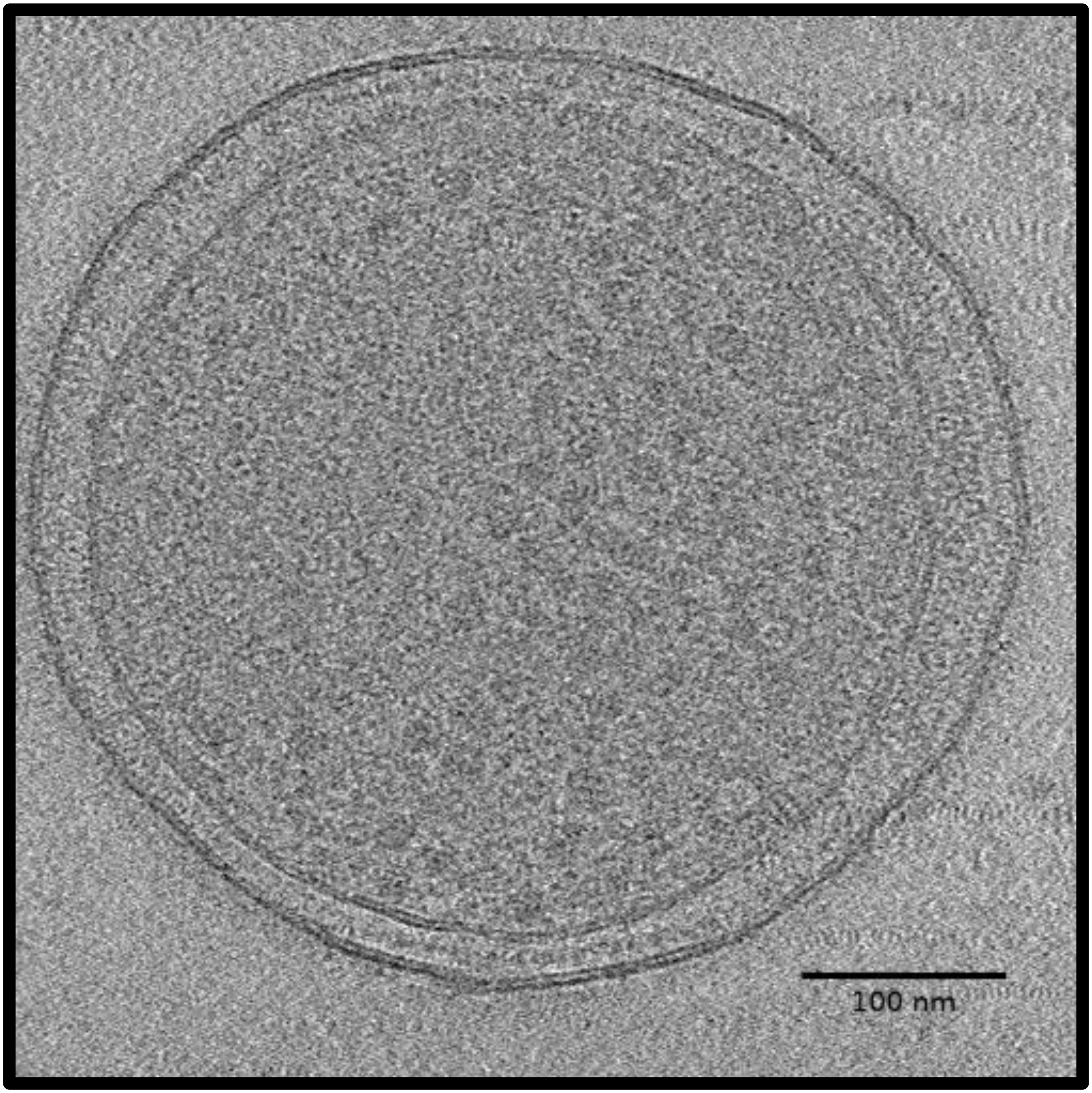

Figure S3. Representative cryo-ET image of AMAX2 minicells. Data were acquired on a Titan Krios transmission electron microscope operated at 300 kV, equipped with a K2 Summit direct electron detector. Tilt series were collected using SerialEM from −60° to +60° in 3° increments with a unidirectional scheme. Images were recorded in counting mode at a nominal magnification of 81,000× (pixel size 12.433 Å). The total accumulated dose was ~150 $e^-/Å^2$, with an energy filter slit width of 20 eV and a defocus range of −2.5 to −5.5 μm.

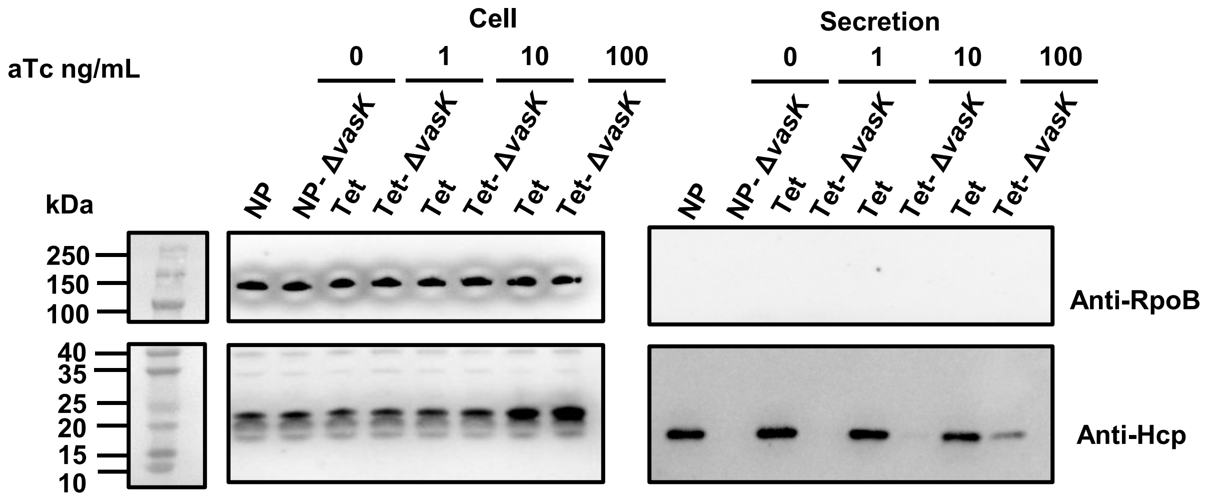

Figure S4. Full uncropped Western blot images for the Hcp secretion assay in AMAX2 carrying pT6S-NP or pT6S-Tet under varying aTc concentrations, corresponding to Fig. 5E–F. For pT6S-Tet strains, expression was induced with 1, 10, or 100 ng/mL anhydrotetracycline. T6SS-deficient controls included *vasK*-deletion strains (pT6S-NP-Δ*vasK* and pT6S-Tet-Δ*vasK*). RpoB was used as a loading and intracellular protein control.

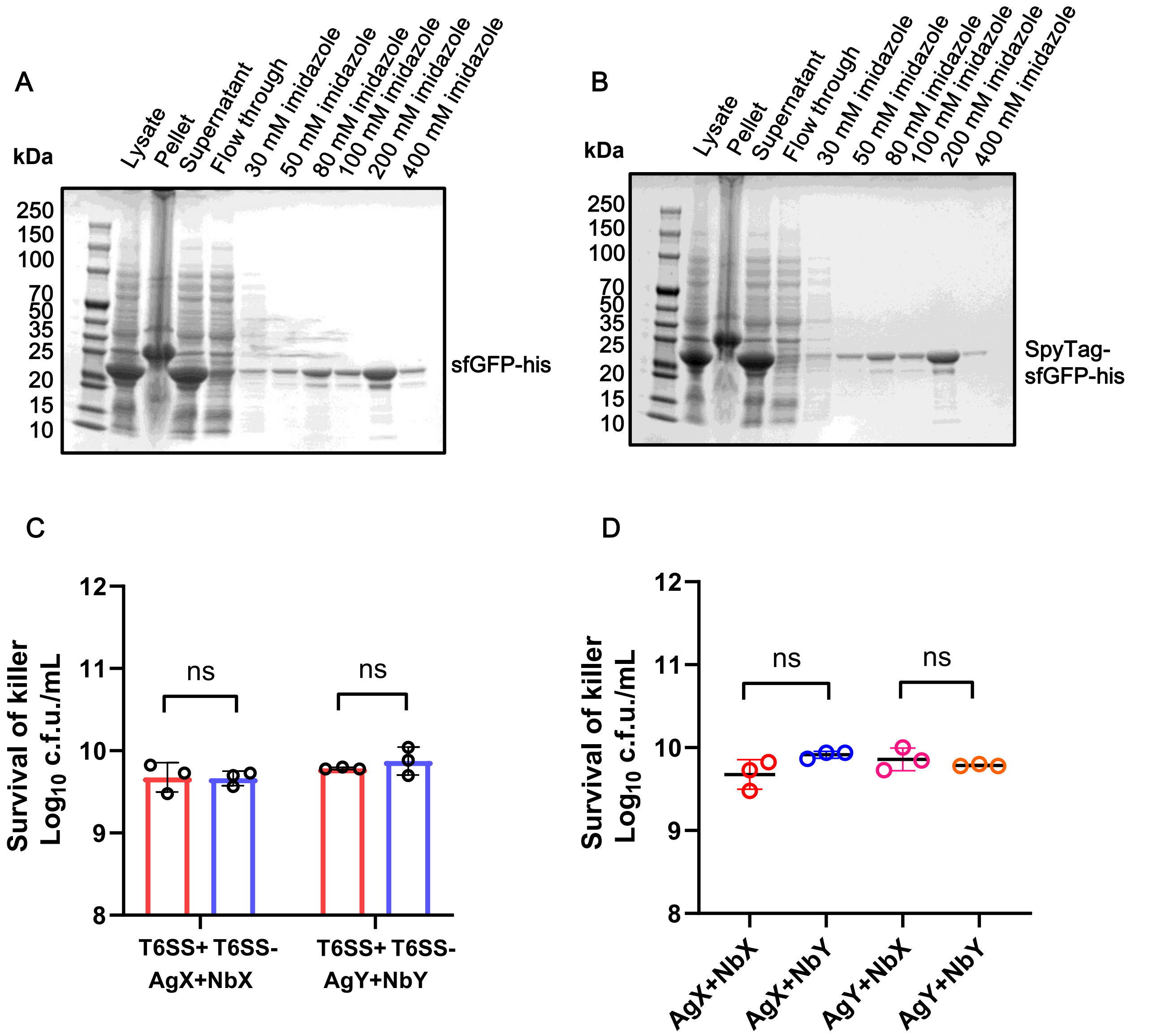

Figure S5. Survival of AMAX2 expressing Nb competed with *E. coli* expressing Ag in liquid coculture. (A–B) Coomassie Brilliant Blue–stained gels of purified sfGFP (A), SpyTag-sfGFP (B). *E. coli* BL21(DE3) cells expressing sfGFP-6×His and SpyTag-sfGFP-6×His from pET22b vectors were cultured at 16 °C for 14 h. Ten microliters of each purified protein sample were loaded per well and separated by electrophoresis using a 12 – 20% denaturing SDS-PAGE gel at 150 V for 40 min. (C) Survival of AMXA2 harboring pT6S-NP (T6SS+) or pT6S-NP-Δv*asK* (T6SS+) expressing Nb competed with *E. coli* expressing cognate Ag following 8-hour competition in liquid LB coculture. (D) Survival of

AMAX2 harboring pT6S-NP expressing Nb competed with *E. coli* expressing Ag following 8-hour competition in liquid LB coculture. For C-D statistical significance was determined using two-way ANOVA followed by Sidak's multiple comparison test (C) or a two-tailed Student's *t*-test (D). ns, not significant.

Tabel S1. List of deleted genes in AMAX1 to generate AMAX2.

Name	Function	Uniprot Number	References
exoA	exotoxin	K1JNX2	(1, 2)
gcat	lipase	K1JKH5	(3)
mepA	metalloprotease	K1JBV9	(4)
aspA	serine protease	K1J282	(5)
ahpB	elastase	K1J276	(6)
lpxM1	lipid A myristoyltransferase	K1JNE6	(7)

Table S2. Codon usage table of AMAX2

Codon	Frequency per thousands	Codon	Frequency per thousands	Codon	Frequency per thousands	Codon	Frequency per thousands
TTT	9.98	TCT	2.82	TAT	10.07	TGT	2.40
TTC	26.08	TCC	15.64	TAC	16.12	TGC	8.66
TTA	1.37	TCA	2.21	TAA	0.99	TGA	1.74
TTG	8.94	TCG	7.47	TAG	0.32	TGG	14.88
CTT	4.10	CCT	3.27	CAT	6.26	CGT	8.78
CTC	27.61	CCC	18.64	CAC	16.97	CGC	31.64
CTA	1.46	CCA	2.73	CAA	7.00	CGA	1.97
CTG	77.63	CCG	22.33	CAG	41.52	CGG	13.26
ATT	6.79	ACT	3.24	AAT	6.47	AGT	4.04
ATC	40.16	ACC	37.15	AAC	23.80	AGC	23.51
ATA	3.09	ACA	2.15	AAA	8.91	AGA	1.63
ATG	25.54	ACG	5.23	AAG	29.45	AGG	2.13
GTT	4.23	GCT	6.64	GAT	22.25	GGT	13.95
GTC	20.15	GCC	66.23	GAC	28.80	GGC	45.09
GTA	4.60	GCA	7.06	GAA	16.90	GGA	3.46
GTG	38.95	GCG	25.19	GAG	42.03	GGG	16.33

Table S3. Strains used in this study.

Strain	Description	Source
E. coli pir1	Strain used for cloning	Invitrogen
E. coli WM6026	Strain used for conjugation	(8)
E. coli DH5α	Strain used for cloning	NEB
E. coli T-fast	Strain used for cloning	TIANGEN
E. coli BL21(DE3)	Strain used for protein expression	Lab stock
E. coli OP50	Strain used to feed nematodes	(9)

Aeromonas dhakensis SSU	Pathogenic control in biosafety assay	Lab stock
AMAX1	*Aeromonas* sp. isolate engineered for protein production	(10)
AMAX2		This study
Pseudomonas aeruginosa PA14	Pathogenic control used in nematode model	(11)

Table S4. Plasmids used in this study.

	Plasmid	Description	Source
pDS132		Suicidal conjugation vector for all chromosomal allelic changes	(12)
	pDS132-dexoA	Suicidal vector to construct AMAX chromosomal Δ*exoA*	This study
	pDS132-dGCAT	Suicidal vector to construct AMAX chromosomal Δ*GCAT lipase*	This study
	pDS132-dmepA	Suicidal vector to construct AMAX chromosomal Δ*mepA*	This study
	pDS132-daspA	Suicidal vector to construct AMAX chromosomal Δ*aspA*	This study
	pDS132-dahpB	Suicidal vector to construct AMAX chromosomal Δ*ahpB*	This study
	pDS132-dlpxM1	Suicidal vector to construct AMAX chromosomal Δ*lpxM1*	This study
	pDS132-dminCDE	Suicidal vector to construct AMAX chromosomal Δ*minCDE*	Lab stock
pBAD	pBAD24kan	Arabinose inducible expression plasmid, kanamycin resistance	Lab stock
	pBAD18cm	Arabinose inducible expression plasmid, chloramphenicol resistance	Lab stock
	pBAD18cm-FtsZ	Arabinose inducible plasmid to express FtsZ	This study
	pBAD24kan-Neae-SpyCatcher-3V5	Arabinose inducible plasmid to express Neae-SpyCatcher-3V5	This study
	pBAD24kan-Neae-SpyTag-3V5	Arabinose inducible plasmid to express Neae-SpyTag-3V5	This study
pET	pET28a-sfGFP-6His	Plasmid to purify sfGFP	Lab stock
	pET22b-sfGFP-His	Plasmid to purify sfGFP	This study
	pET22b-sfGFP-SpyCatcher-His	Plasmid to purify sfGFP-SpyCatcher	This study
	pET22b-SpyTag-sfGFP-His	Plasmid to purify SpyTag-sfGFP	This study
	pET22b-5×AGA-sfGFP-His	Plasmid to express rare codon plus sfGFP	This study
	pET22b-5×AGG-sfGFP-His	Plasmid to express rare codon plus sfGFP	This study

	pET22b-5×AUA-sfGFP-His	Plasmid to express rare codon plus sfGFP	This study
	pET22b-5×CGG-sfGFP-His	Plasmid to express rare codon plus sfGFP	This study
	pET22b-5×CUA-sfGFP-His	Plasmid to express rare codon plus sfGFP	This study
pPSV37	pPSV37-sfGFP	IPTG inducible plasmid to express sfGFP	Lab stock
p15A	p15A-sfGFP	Anhydrotetracycline inducible plasmid to express sfGFP	Lab stock
	p15A-T6S-NP	Plasmid to constitutively express active T6SS	(13)
	p15A-T6S-NP-Δ*vasK*	Plasmid to constitutively express inactive T6SS	(13)
	p15A-T6S-Tet	Anhydrotetracycline inducible plasmid to express T6SS	(13)
	p15A-T6S-Tet-Δ*vasK*	Anhydrotetracycline inducible plasmid to inactive T6SS	(13)
pCas9		Plasmid to express Cas9 and λ-Red recombination system	Lab stock
pCRISPR	pCRISPR-lacZ	Plasmid express sgRNA targeting *lacZ* and with repair template	This study

Table S5. Primers used in this study.

Primer	Sequence (5'-3')	Description
pDS132-hifi-f	cgatccttttaacccatcac	Forward primer to amplify pDS132 vector
pDS132-hifi-r	cttctagaggtaccgcatgc	Reverse primer to amplify pDS132 vector
pDS132-f	tgttgcatgggcataaagttgc	Forward confirmation primer of pDS132 vector
pDS132-r	acggctgacatgggaattcc	Reverse confirmation primer of pDS132 vector
pDS132-dexoA-KO1	gtgatgggttaaaaaggatcgatcaacagcggctacggc	Forward primer to amplify the upstream homologs arm for constructing in-frame deletion of *exoA*
pDS132-dexoA-KO2	tgtgtttgtctgcatgtgatttcc	Reverse primer to amplify the upstream homologs arm for constructing in-frame deletion of *exoA*
pDS132-dexoA-KO3	atcacatgcagacaaacacacgcgacgaactctgaatcg	Forward primer to amplify the downstream homologs arm for constructing in-frame deletion of *exoA*
pDS132-dexoA-KO4	gcatgcggtacctctagaagctttatcgccctcaacaagcc	Reverse primer to amplify the downstream homologs arm for constructing in-frame deletion of *exoA*
pDS132-dexoA-KO5	gctgctgataagctgctttgg	Forward confirmation primer of *exoA*
pDS132-dexoA-KO6	ggtgggggatcaggtgtttg	Reverse confirmation primer of *exoA*
pDS132-dGCAT-KO1	gtgatgggttaaaaaggatcgagttaatcgaggcgggtctg	Forward primer to amplify the upstream homologs arm for constructing in-frame deletion of *GCAT lipase*
pDS132-dGCAT-KO2	ttcctcgcccattgatgacaa	Reverse primer to amplify the upstream homologs arm for constructing in-frame deletion of *GCAT lipase*

pDS132-dGCAT-KO3	tgtcatcaatgggcgaggaaaagctgttttgccatcatgcag	Forward primer to amplify the downstream homologs arm for constructing in-frame deletion of *GCAT lipase*
pDS132-dGCAT-KO4	gcatgcggtacctctagaaggtcatctcggcctacaacg	Reverse primer to amplify the downstream homologs arm for constructing in-frame deletion of *GCAT lipase*
pDS132-dGCAT-KO5	ctccttatgatgggtcgggc	Forward confirmation primer of *GCAT lipase*
pDS132-dGCAT-KO6	gcagaccgtctatctggtgg	Reverse confirmation primer of *GCAT lipase*
pDS132-dmepA-KO1	gtgatgggttaaaaaggatcgaaaggtaaggacacaccccc	Forward primer to amplify the upstream homologs arm for constructing in-frame deletion of *mepA*
pDS132-dmepA-KO2	gtctcatgcttgccaccaccccgttgtggcggttgacca	Reverse primer to amplify the upstream homologs arm for constructing in-frame deletion of *mepA*
pDS132-dmepA-KO3	ggtggtggcaagcatgaga	Forward primer to amplify the downstream homologs arm for constructing in-frame deletion of *mepA*
pDS132-dmepA-KO4	gcatgcggtacctctagaagccgagctgatagccgtact	Reverse primer to amplify the downstream homologs arm for constructing in-frame deletion of *mepA*
pDS132-dmepA-KO5	cgtcatccggtacgacagat	Forward confirmation primer of *mepA*
pDS132-dmepA-KO6	gcagtacgccgttgtctttg	Reverse confirmation primer of *mepA*
pDS132-daspA-KO1	gtgatgggttaaaaaggatcgaaaccgcccatctgcattct	Forward primer to amplify the upstream homologs arm for constructing in-frame deletion of *aspA*
pDS132-daspA-KO2	cgcttggctttgtattgcgggcccagcaggtcttcttcaa	Reverse primer to amplify the upstream homologs arm for constructing in-frame deletion of *aspA*
pDS132-daspA-KO3	ccgcaatacaaagccaagcg	Forward primer to amplify the downstream homologs arm for constructing in-frame deletion of *aspA*
pDS132-daspA-KO4	gcatgcggtacctctagaaggcgatgaagatccagaccga	Reverse primer to amplify the downstream homologs arm for constructing in-frame deletion of *aspA*
pDS132-daspA-KO5	ttactccttgcgctcgaact	Forward confirmation primer of *aspA*
pDS132-daspA-KO6	ggcccactttaccggagatg	Reverse confirmation primer of *aspA*
pDS132-dahpB-KO1	gtgatgggttaaaaaggatcgctgctggatgtggtcaatcg	Forward primer to amplify the upstream homologs arm for constructing in-frame deletion of *ahpB*
pDS132-dahpB-KO2	gacggatgaacaaagtctatctcaaggccagctactgatca	Reverse primer to amplify the upstream homologs arm for constructing in-frame deletion of *ahpB*
pDS132-dahpB-KO3	gcagaccgtctatctggtgg	Forward primer to amplify the downstream homologs arm for constructing in-frame deletion of *ahpB*
pDS132-dahpB-KO4	gcatgcggtacctctagaagggctgtagtgaacgtgatcca	Reverse primer to amplify the downstream homologs arm for constructing in-frame deletion of *ahpB*

pDS132-dahpB-KO5	atagtggtagccgacaactgg	Forward confirmation primer of *ahpB*
pDS132-dahpB -KO6	atagtggtagccgacaactgg	Reverse confirmation primer of *ahpB*
pDS132-lpxM1-KO1	tgatgggttaaaaaggatcgcttgggatggccgaagaagg	Forward primer to amplify the upstream homologs arm for constructing in-frame deletion of *lpxM1*
pDS132-lpxM1-KO2	cagccgctctcctgatgag	Reverse primer to amplify the upstream homologs arm for constructing in-frame deletion of *lpxM1*
pDS132-lpxM1-KO3	gctcatcaggagagcggctggaaagggagctgcattgtattcat	Forward primer to amplify the downstream homologs arm for constructing in-frame deletion of *lpxM1*
pDS132-lpxM1-KO4	gcatgcggtacctctagaaggtggtctccaaggcggtc	Reverse primer to amplify the downstream homologs arm for constructing in-frame deletion of *lpxM1*
pDS132-lpxM1-KO5	catagcaggcgaggatggg	Forward confirmation primer of *lpxM1*
pDS132-lpxM1-KO6	gatctggctcaccccgtg	Reverse confirmation primer of *lpxM1*
pDS132-minCDE-KO1	tgatgggttaaaaaggatcgcagctgccccttgaagaagg	Forward primer to amplify the upstream homologs arm for constructing in-frame deletion of *minCDE*
pDS132-minCDE-KO2	cttcttgtcatcgcgctcaaccatgaatc	Reverse primer to amplify the upstream homologs arm for constructing in-frame deletion of *minCDE*
pDS132-minCDE-KO3	ttgagcgcgatgacaagaagggatgagcctgac	Forward primer to amplify the downstream homologs arm for constructing in-frame deletion of *minCDE*
pDS132-minCDE-KO4	gcatgcggtacctctagaagtgcttcacctcgaccacctt	Reverse primer to amplify the downstream homologs arm for constructing in-frame deletion of *minCDE*
pDS132-minCDE-KO5	actgcacctgtcccat	Forward confirmation primer of *minCDE*
pDS132-minCDE-KO6	gcatccatgtcgaagccgtt	Reverse confirmation primer of *minCDE*
pBAD24-V5-hifi-f	ggtaaacctattcctaatcctctcctt	Forward primer to amplify pBAD24-3V5 vector
pBAD24-hifi-r	ggtacctcctgctagcccaaa	Reverse primer to amplify pBAD24-3V5 vector
pBAD18cm-hifi-f	tacccggggatcctctagagt	Forward primer to amplify pBAD18vector
pBAD18cm-hifi-r	cgtttcctcctccgagctcgaattcgctagc	Reverse primer to amplify pBAD18 vector
pBAD-f	agtccacattgattatttgcacgg	Forward confirmation primer of pBAD vector
pBAD-r	ttcacttctgagttcggcatgg	Reverse confirmation primer of pBAD vector
Neae-SpyTag-f	ttgggctagcaggaggtaccatgattactcatggttgttataccc	Forward primer to amplify Neae-SpyTag to construct pBAD24-Neae-SpyTag-3V5
Neae-SpyTag-f	ggattaggaataggtttacctttggtcggcttataggcatccaccatcacgatatgggctctagtcgcaccatcaaaaaatataacc	Reverse primer to amplify Neae-SpyTag to construct pBAD24-Neae-SpyTag-3V5

Neae-r	tctagtcgcaccatcaaaaaatataacc	Reverse primer to amplify Neae to construct pBAD24-Neae-SpyCatcher-3V5
SpyCatcher-f	tttttgatggtgcgactagagcggccgcaggaggaggagttgataccctgagcggcct	Forward primer to amplify SpyCatcher to construct pBAD24-Neae-SpyCatcher-3V5
SpyCatcher-r	ggattaggaataggtttaccaatatgtgcgtcaccttttgtgg	Reverse primer to amplify SpyCatcher to construct pBAD24-Neae-SpyCatcher-3V5
FtsZ-f	cgagctcggaggaggaaacgatgtttgaatttatggatagccaca	Forward primer to amplify FtsZ to construct pBAD18-FtsZ
FtsZ-f	actctagaggatccccgggtattagtcagcttgcttgcgca	Reverse primer to amplify FtsZ to construct pBAD18-FtsZ
pCRISPR-confrim-f	agacgaagaatccatggcgg	Forward confirmation primer for pCRISPR
pCRISPR-confrim-r	aagcttctgaatggcgggag	Reverse confirmation primer for pCRISPR
lacZ-sgRNA-f	gtggttcggctccgtcgaggtcga	Forward primer with sgRNA targeting *lacZ*
lacZ-sgRNA-r	aaactcgacctcgacggagccgaa	Reverse primer with sgRNA targeting *lacZ*
lacZ-repair-f	ccatggtctagagggtttcgatgacatcatctatgc	Forward primer to amplify the repair templated of *lacZ*
lacZ-repair-r	gaaaagtctcgagagcgccttcaccccctt	Reverse primer to amplify the repair templated of *lacZ*
pET22b-f	aagcttgcggccgcactcga	Forward primer to amplify pET22b vector
pET22b-r	catatgtatatctccttcttaaagttaaacaaaattatttctagag	Reverse primer to amplify pET22b vector
pETDuet-f	cacgatgcgtccggcgtagagg	Forward confirmation primer for pET plasmid
pETDuet-r	ggttatgctagttattgctcagcggt	Reverse confirmation primer for pET plasmid
pET22b-sfgfp-AGA-f	aagaaggagatatacatatgagaagaagaagaagatctaaaggtgaagaactgttcacc	Forward primer to amplify 5×AGA-sfGFP
pET22b-sfgfp-AGG-f	aagaaggagatatacatatgaggaggaggaggaggtctaaaggtgaagaactgttcacc	Forward primer to amplify 5×AGG-sfGFP
pET22b-sfgfp-AUA-f	aagaaggagatatacatatgataataataataatatctaaaggtgaagaactgttcacc	Forward primer to amplify 5×AUA-sfGFP
pET22b-sfgfp-CGG-f	aagaaggagatatacatatgcggcggcggcggcggtctaaaggtgaagaactgttcacc	Forward primer to amplify 5×CGG-sfGFP
pET22b-sfgfp-CUA-f	aagaaggagatatacatatgctactactactactatctaaaggtgaagaactgttcacc	Forward primer to amplify 5×CUA-sfGFP
pET22b-sfgfp-r	tcgagtgcggccgcaagcttttgtagagctcatccatgccg	Reverse primer to amplify sfGFP

References:

1. Mosser T, Talagrand-Reboul E, Colston SM, Graf J, Figueras MJ, Jumas-Bilak E, Lamy B. 2015. Exposure to pairs of *Aeromonas* strains enhances virulence in the *Caenorhabditis elegans* infection model. Front Microbiol 6.

2. Ponnusamy D, Kozlova E V., Sha J, Erova TE, Azar SR, Fitts EC, Kirtley ML, Tiner BL, Andersson JA, Grim CJ, Isom RP, Hasan NA, Colwell RR, Chopra AK. 2016. Cross-talk among flesh-eating *Aeromonas hydrophila* strains in mixed infection leading to necrotizing fasciitis. Proceedings of the National Academy of Sciences 113:722–727.

3. Lee KK, Ellis AE. 1990. Glycerophospholipid:cholesterol acyltransferase complexed with lipopolysaccharide (LPS) is a major lethal exotoxin and cytolysin of *Aeromonas salmonicida*: LPS stabilizes and enhances toxicity of the enzyme. J Bacteriol 172:5382–5393.

4. Yu HB, Zhang YL, Lau YL, Yao F, Vilches S, Merino S, Tomas JM, Howard SP, Leung KY. 2005. Identification and characterization of putative virulence genes and gene clusters in *Aeromonas hydrophila* PPD134/91. Appl Environ Microbiol 71:4469–77.

5. Vipond R, Bricknell IR, Durant E, Bowden TJ, Ellis AE, Smith M, MacIntyre S. 1998. Defined deletion mutants demonstrate that the major secreted toxins are not essential for the virulence of *Aeromonas salmonicida*. Infect Immun 66:1990–1998.

6. Cascón A, Yugueros J, Temprano A, Sánchez M, Hernanz C, Luengo JM, Naharro G. 2000. A major secreted elastase is essential for pathogenicity of *Aeromonas hydrophila*. Infect Immun 68:3233–41.

7. Somerville JE, Cassiano L, Darveau RP. 1999. *Escherichia coli* msbB gene as a virulence factor and a therapeutic target. Infect Immun 67:6583–90.

8. Blodgett JA V, Thomas PM, Li G, Velasquez JE, van der Donk WA, Kelleher NL, Metcalf WW. 2007. Unusual transformations in the biosynthesis of the antibiotic phosphinothricin tripeptide. Nat Chem Biol 3:480–485.

9. Brenner S. 1974. The genetics of *Caenorhabditis elegans*. Genetics 77:71–94.

10. Tang M-X, Meng P-F, Huang R-L, Zheng X, Liang C-C, Pu X, Wang C, Zhao Y, Zhang Y-Q, Liang J-X, Yan Y-X, Xiao Y, An Y, Liang X, Song Y, Qu J, Yu B, Xia Y, Dong T. 2025. *Aeromonas* spp. as a fast-growing high-performance chassis for protein production. Appl Environ Microbiol 91.

11. Tan MW, Mahajan-Miklos S, Ausubel FM. 1999. Killing of *Caenorhabditis elegans* by *Pseudomonas aeruginosa* used to model mammalian bacterial pathogenesis. Proc Natl Acad Sci U S A 96:715–720.

12. Philippe N, Alcaraz JP, Coursange E, Geiselmann J, Schneider D. 2004. Improvement of pCVD442, a suicide plasmid for gene allele exchange in bacteria. Plasmid 51:246–255.

13. Yang C, Tong-Tong P, Xiaoye L, Hao L, Hao-Yu Z, Tao D. 2022. Heterologous assembly of the type VI secretion system empowers laboratory *Escherichia coli* with antimicrobial and cell penetration capabilities. Appl Environ Microbiol 88:e01305-22.
