# Supplementary material for: A programmable Aeromonas chassis, AMAX2, for advanced biomanufacturing
Source: Appl Environ Microbiol. 2026 May 18;92(6):e00231-26. doi: 10.1128/aem.00231-26 (PMC13274425; doi:10.1128/aem.00231-26)
Supplement: Supplemental legends — Descriptive legends for Movies S1 and S2. [file aem.00231-26-s0003.pdf]

Supplementary material file

A programmable *Aeromonas* chassis AMAX2 for advanced biomanufacturing

Ming-Xuan Tang, Yu-Zhao Liu, Jia-Xin Liang, Ruo-Lin Huang, Xuepiao Pu, Chen-Chen Liang, Zi-Yu Tang, Tong-Tong Pei, Ya-Jie Zhao, Hao-Yu Zheng, Tingting Zhang, Zixian Wu, Ying An, Xiaoye Liang, Xue Liu, Tao Dong

Movie S1. Time-lapse movie showing VipA-sfGFP localization in AMAX2 with pT6S-NP. Imaging was performed at 37 °C using a Nikon Ti2-E inverted microscope. Images were acquired every 10 s for 5 min, and a representative 20 × 20 μm field is shown.

Movie S2. Time-lapse movie showing VipA-sfGFP localization in AMAX2 with pT6S-Tet. Imaging was performed at 37 °C using a Nikon Ti2-E inverted microscope. Images were acquired every 10 s for 5 min, and a representative 20 × 20 μm field is shown.
